# Redox-endocrine triad in PCOS: can vitamin D, myo-inositol, and melatonin synergize as bioactive cocktails?

**DOI:** 10.3389/fendo.2026.1825853

**Published:** 2026-04-28

**Authors:** Sumayyah Subakathulla, Norwin Manoj, Azima Muzzammil Patanwala, Prasanna Appiya Premvignesh, Yamen Maher Alobaid, Ankit Majie, Bapi Gorain, Sulagna Dutta, Pallav Sengupta, Israel Maldonado Rosas, Shubhadeep Roychoudhury

**Affiliations:** 1 Department of Biomedical Sciences, College of Medicine, Gulf Medical University, Ajman, United Arab Emirates; 2 Department of Pharmaceutical Sciences and Technology, Birla Institute of Technology, Mesra, Ranchi, India; 3 Basic Medical Sciences Department, College of Medicine, Ajman University, Ajman, United Arab Emirates; 4 Centre of Medical and Bio-Allied Health Sciences Research, Ajman University, Ajman, United Arab Emirates; 5 Citmer Reproductive Medicine, Mexico City, Mexico; 6 Department of Life Science and Bioinformatics, Assam University, Silchar, India

**Keywords:** insulin resistance, nutraceutical, oxidative stress, polycystic ovary syndrome, redox-endocrine axis

## Abstract

Polycystic ovary syndrome (PCOS) is a heterogeneous endocrine–metabolic disorder in which reproductive dysfunction coexists with insulin resistance, chronic low-grade inflammation, and heightened oxidative stress (OS). Increasing evidence indicates that these abnormalities are not independent phenomena but components of a self-perpetuating redox-endocrine network that sustains hyperandrogenism, anovulation, and metabolic impairment. This review critically synthesizes experimental, translational, and clinical data to examine whether vitamin D, myo-inositol, and melatonin, three widely used but often studied in isolation bioactives, can act synergistically as a mechanistically coherent ‘bioactive cocktail’ in PCOS. Vitamin D modulates inflammatory tone and steroidogenic signaling through vitamin D receptor-dependent transcription and immune–metabolic crosstalk; myo-inositol restores insulin signaling via inositolphosphoglycan second-messenger pathways, thereby attenuating hyperinsulinemia-driven androgen excess; and melatonin exerts pleiotropic effects on mitochondrial function, circadian regulation, and redox balance. Therefore, these agents converge on shared molecular hubs, including NF-κB, Nrf2, PI3K/Akt, and AMPK, linking OS reduction with endocrine and metabolic recalibration. The review further integrates emerging insights into gut microbiota-adipokineinteractions, highlighting how dysbiosis and altered adipokine profiles amplify oxidative and hormonal disturbances, and how these bioactives may counteract such system-level disruptions. While existing clinical trials report improvements in ovulatory function, insulin resistance indices, and OS biomarkers, outcomes remain heterogeneous due to differences in dosing, duration, and phenotype stratification. We propose a redox-guided, phenotype-aware framework for future trials, emphasizing biomarker-anchored outcomes and systems-level integration. If validated, combined vitamin D, myo-inositol, and melatonin supplementation may represent a precision nutraceutical strategy that targets the pathogenic core of PCOS rather than its isolated clinical manifestations.

## 1 Introduction

Polycystic ovary syndrome (PCOS) represents one of the most complex endocrine-metabolic disorders affecting women of reproductive age, characterized by a collection of reproductive, metabolic, and psychological manifestations (1, 2). According to the World Health Organization (WHO), PCOS is the leading cause of infertility and anovulation around the globe. This condition also affects an individual’s long-term physical and emotional well-being. Approximately 6-13% of female individuals in their reproductive age are affected by PCOS, and out of these, 70% remain undiagnosed (3). Around 50% of women remain unaware of this condition, contributing to delayed diagnosis (4). A study published in 2025 reported disability as a serious consequence of PCOS. The global prevalence of PCOS increased from 36.7 to 69.5 million between 1990 and 2021. The incidence of PCOS increased from 1.5 to 2.3 million during this period. While years lived with disability increased from 323,799 to 607,757 (5).

Beyond the classic triad of hyperandrogenism (HA), ovulatory dysfunction, and polycystic ovarian morphology, PCOS extends into domains of insulin resistance, dyslipidemia, chronic inflammation, and heightened oxidative stress (OS) (6, 7). This intricate network of abnormalities positions PCOS not merely as a reproductive disorder but as a systemic condition reflecting redox-endocrine disequilibrium. Emerging evidence underscores that OS is not a mere byproduct of metabolic imbalance but a central driver that disrupts insulin signaling, amplifies androgen biosynthesis, and impairs folliculogenesis (8, 9). Concurrently, hormonal dysregulation, particularly involving insulin, androgens, and gonadotropins, feeds back into redox instability, creating a vicious cycle of endocrine and metabolic dysfunction (10). Understanding the intricate molecular details of the redox-endocrine triad is key to developing new therapeutics. A study of 80 pregnant individuals with PCOS demonstrated significant elevations in hormones such as thyroid-stimulating hormone (TSH), luteinizing hormone (LH), follicle-stimulating hormone (FSH), and insulin, as well as an OS marker, malondialdehyde (MDA), indicating the involvement of both endocrine and redox systems (11). Hyperandrogenism is characterized by insulin resistance and inflammation, which create a detrimental feedback loop that produces more reactive oxygen species (ROS) (12). The increasing ROS targets the downregulation of growth hormone (GH), which further inhibits the PI3K/Akt signaling pathway, leading to the apoptosis of granulosa cells. Thus, these studies highlight the importance of ROS and hormonal imbalance in PCOS (13).

Conventional therapeutic strategies, though effective in symptom control, remain largely palliative, targeting isolated components of this multifactorial syndrome (14). Pharmacological options such as metformin, anti-androgen agents, or oral contraceptive pills (OCPs) often bring limited long-term benefits. They may not address the underlying molecular chaos of redox and endocrine crosstalk (15). Additionally, the use of these agents has several reported severe adverse effects, such as pregnancy loss, risks of malignancy, liver toxicity, thromboembolic episodes, and lactic acidosis (16). Low-dose variants of OCPs have been observed with an increased risk of myocardial infarction and cardiovascular arterial disease (17). Off-label use of United States Food and Drug Administration (US-FDA)-approved drugs, such as medroxyprogesterone acetate, norgestimate, ethinyl estradiol, and eflornithine, has shown side effects, such as breast tenderness, thrombosis, venous thromboembolism, mood swings, menstrual irregularity, migraine, and topical irritation (18). This therapeutic gap has prompted growing interest in bioactive nutraceuticals, natural molecules capable of restoring redox balance and hormonal harmony through multi-level actions (19). Among them, vitamin D, myo-inositol (MI), and melatonin have independently demonstrated promising roles as supplements in improving insulin sensitivity, endometrial thickness, follicular health, embryo/egg quality, oxidative resilience, sleep cycle, mood, and regularizing menstrual cycles in women (20–22). Currently, these three therapeutic agents are generally recognized as safe (GRAS), with vitamin D approved by the FDA as a food fortification agent (23, 24).

Thus, this review critically examines the triadic potential of these agents, not as standalone supplements but as a synergistic bioactive cocktail targeting the redox-endocrine axis. By integrating evidence from molecular, clinical, and translational studies, it seeks to elucidate whether the concerted modulation of OS, mitochondrial dynamics, and hormonal signaling by vitamin D, MI, and melatonin can redefine adjunctive therapy in PCOS.

## 2 Redox biology and endocrine dysregulation in PCOS

### 2.1 OS pathways in PCOS: sources of ROS and antioxidant defense impairment

Emerging evidence identifies PCOS as a disorder of redox dyshomeostasis, contributing to its complex reproductive, endocrine, and metabolic manifestations (12, 25). In women affected by PCOS, multiple interconnected molecular and cellular pathways give rise to elevated reactive oxygen species (ROS) (26). Key contributors include mitochondrial inefficiency, chronic inflammation, androgen excess, and metabolic dysregulation (hyperglycemia, dyslipidemia), all of which may challenge and eventually overwhelm the antioxidant defense network (27, 28).

Among endogenous sources, mitochondrial electron transport chain (ETC) inefficiency stands out as a dominant ROS generator in PCOS (29). Recent metabolomic profiling of follicular fluid from women with PCOS, even those with normal weight, demonstrated altered mitochondrial and glycolytic intermediates, specifically decreased lactate and formate and elevated citrate, indicative of ETC fluctuations and increased oxidative burden in the follicular milieu (30, 31). Simultaneously, chronic low-grade inflammation increases ROS production. This occurs as heightened inflammatory signaling cascades trigger ROS-generating enzymes like NADPH oxidase (NOX), which, in turn, intensifies oxidative damage (32). In a recent case-control study of hyperandrogenic, insulin-resistant PCOS patients, strong correlations between OS biomarkers and inflammatory mediators were observed, underscoring the crosstalk between inflammation and redox imbalance (33).

Androgen excess emerges not only as a hormonal hallmark of PCOS but also as a direct ROS instigator (34, 35). Animal experiments reveal that dihydrotestosterone (DHT) can trigger ovarian ferroptosis, an iron-dependent, ROS-driven cell death process, by enhanced ferritinophagy. That pharmacological suppression of ferroptosis can attenuate PCOS features, including HA, ovulatory failure, and glucose intolerance (36). Parallel metabolic disturbances, elevated glucose, dyslipidemia, and increased free fatty acids, complicate this picture further by promoting mitochondrial overload, non-enzymatic glycation, and lipid peroxidation (LPO) (37, 38). Recently, iron-mediated LPO has gained attention, as altered expression of iron-regulatory genes in PCOS may facilitate catalytic ROS formation within lipid membranes, adding an extra layer of oxidative risk (39). In addition to mitochondrial and iron-derived ROS, impaired nitric oxide (NO) signaling constitutes another pivotal source of redox disruption in PCOS. Elevated levels of asymmetric dimethylarginine (ADMA) frequently inhibit nitric oxide synthase (NOS) activity, thereby diminishing NO bioavailability and steering the cellular milieu from nitrosative equilibrium toward oxidative injury (40, 41). This shift has been implicated in disrupted follicular dynamics, ovulatory dysfunction, and vascular endothelial stress (40).

While ROS sources increase, antioxidant defenses in PCOS are often found depleted (42). Clinical investigations have consistently documented significantly lower total antioxidant capacity (TAC) in serum and plasma across diverse PCOS populations, spanning adolescents, non-obese, and obese women alike (28, 43). A cross-sectional study in non-obese adolescents even reported diminished TAC after adjusting for body mass index (44). The glutathione (GSH) redox system and its associated enzymes, glutathione peroxidase (GPx) and glutathione reductase (GR), are commonly compromised in both serum and follicular fluid of PCOS patients. Lower GSH levels correlate with poorer oocyte and embryo quality in IVF settings (45). Studies examining first-retrieved follicles reveal that reduced GPx and GR activities, along with decreased GSH, are associated with fewer mature oocytes and lower numbers of high-grade embryos (46). Likewise, classic antioxidant enzymes, such as superoxide dismutase (SOD), exhibit reduced activity in PCOS. A UAE-based case-control study reported lower SOD activity, increased LPO, and elevated homocysteine levels in women with PCOS, with these findings correlating with age, body mass, and disease duration (47). Non-enzymatic antioxidants, including GSH itself, as well as vitamins C and E, are also compromised in a 2024 Iraqi cohort study. Women with PCOS exhibited significantly lower GSH and vitamin C concentrations and markedly elevated malondialdehyde (MDA), a marker of LPO (48). An imbalance between ROS generation and antioxidant defenses leads to the accumulation of oxidative damage, wherein LPO compromises membrane integrity, protein carbonylation alters enzymatic and structural proteins, and oxidative DNA lesions undermine genomic stability (7, 49). These molecular impacts hamper insulin signaling by oxidative modification of insulin receptor substrates (IRS) and downstream kinases, thereby aggravating insulin resistance (IR) (25). Elevated ROS also interferes with the steroidogenic machinery of theca and granulosa cells, impairing follicular maturation and fostering follicular arrest (50). Experimental models support this link that suppression of ROS generation or boosting antioxidant resilience can partially rescue reproductive dysfunction in PCOS models (51). Thus, the oxidative milieu of PCOS is influenced by multiple overlying ROS sources, including mitochondrial leakage, inflammatory induction, androgenic and iron-driven pathways, and NO signaling dysregulation, while the antioxidant network is evidently weakened. The cumulative oxidative burden inflicts molecular damage that intersects IR and ovarian dysfunction.

### 2.2 Mitochondrial dysfunction, lipid peroxidation, and redox imbalance in oocytes and granulosa cells

The competence of oocytes and their surrounding granulosa cells is fundamentally governed by mitochondrial health, balanced redox homeostasis, and membrane integrity (52, 53). In the context of PCOS, a growing body of molecular, cellular, and clinical evidence underscores that mitochondrial dysfunction and oxidative damage within these cells are central to impaired oocyte maturation, apoptosis, and suboptimal embryonic development outcomes. Mitochondrial impairment in granulosa cells of PCOS patients has been consistently demonstrated across transcriptomic, ultrastructural, and metabolic studies (27, 54). These cells exhibit aberrant mitochondrial morphology, loss of cristae, reduced mitochondrial membrane potential (ΔΨm), diminished ATP synthesis, and dysregulated expression of mitochondrial biogenesis regulators (55). It has been reported that granulosa cells from PCOS women exhibit swollen mitochondria and disrupted ultrastructure, along with reduced expression of SIRT1 and PGC-1α, key regulators of mitochondrial biogenesis and oxidative metabolism. This downregulation of the SIRT1/AMPK/PGC-1α axis appears to integrate metabolic stress with mitochondrial injury, thereby linking systemic metabolic imbalance to local cellular dysfunction (56, 57). Complementary transcriptomic analyses reinforce this finding that genes involved in oxidative phosphorylation and mitochondrial metabolism are downregulated in PCOS granulosa cells compared to normal responders, supporting the hypothesis that defective bioenergetics and redox regulation underlie follicular insufficiency (58). Parallel to granulosa cell dysfunction, oocytes in PCOS (and in corresponding animal models) display notable mitochondrial and redox alterations. Rodent studies consistently report decreased ΔΨm and elevated ROS accumulation in oocytes from PCOS models, while ATP production and mitochondrial complex expression show variable patterns across experimental settings (58, 59). In human oocytes, metabolic tracer studies have revealed increased glucose and pyruvate consumption, likely a compensatory mechanism to offset mitochondrial inefficiency (60). Moreover, a recent report demonstrated significantly reduced mitochondrial DNA (mtDNA) copy numbers in cumulus–oocyte complexes from PCOS women, accompanied by increased oxidative modifications of mtDNA, confirming that mitochondrial integrity within the gamete microenvironment is severely compromised (61).

LPO is an evident indicator of oxidative injury in ovarian cells. The polyunsaturated fatty acids (PUFAs) within mitochondrial and cellular membranes are particularly vulnerable to ROS-mediated peroxidation, yielding cytotoxic byproducts such as MDA and 4-hydroxynonenal (4-HNE) (49, 62). Elevated MDA levels in follicular fluid and granulosa cell lysates from PCOS patients are a consistent observation, often correlating inversely with oocyte quality and embryo development metrics (63). These LPO products disturb membrane fluidity, receptor activity, ion exchange, and mitochondrial electron transport, thereby amplifying ROS generation in a self-perpetuating oxidative cycle (64). Redox disequilibrium in ovarian compartments is further substantiated by alterations in key molecular markers. The reduced-to-oxidized glutathione (GSH/GSSG) ratio, an essential indicator of redox homeostasis, is markedly lower in PCOS follicular fluid and granulosa cell samples, reflecting compromised antioxidant regeneration capacity (7). The availability of NADPH, a vital cofactor for GSH recycling, may be limited due to mitochondrial metabolic restriction or disrupted pentose phosphate pathway flux (65). Moreover, the mitochondrial deacetylase SIRT3, an enzyme essential for maintaining mitochondrial redox balance, has been found deficient in granulosa cells of PCOS patients, contributing to elevated OS, mitochondrial fragmentation, and apoptosis (66). Thus, these molecular imbalances redirect cellular fate from adaptive survival toward oxidative injury and programmed cell death. These mitochondrial and redox abnormalities have profound consequences for ovarian physiology. Reduced ATP availability weakens meiotic spindle formation and chromosome segregation (67); oxidative DNA and protein damage compromise genomic and proteomic stability (68); and peroxidized membranes impair ion gradients and intracellular signaling cascades (69). Simultaneously, increased granulosa cell apoptosis and reduced estrogen synthesis compromise the nurturing microenvironment essential for oocyte development. The result of these cellular injuries manifests clinically as reduced fertilization rates, lower-quality embryos, and increased developmental arrest in PCOS-associated infertility (70).

### 2.3 IR, HA, and inflammation as endocrine correlates of OS

IR, HA, and chronic low-grade inflammation constitute the central endocrine correlates of OS in PCOS (71). These three pathophysiological pillars are intricately linked through a self-perpetuating redox-endocrine loop that amplifies metabolic and reproductive dysfunction. IR, present in up to 70% of women with PCOS irrespective of obesity, is both a cause and consequence of oxidative imbalance. Excess ROS impairs IRS phosphorylation and attenuates phosphatidylinositol 3-kinase (PI3K)/Akt signaling, leading to diminished glucose uptake and hyperinsulinemia (72). The latter, in turn, promotes ovarian theca cell steroidogenesis and suppresses hepatic synthesis of sex hormone-binding globulin (SHBG), thus increasing free testosterone levels and driving HA (72).

Androgen excess further exacerbates OS by stimulating nicotinamide adenine dinucleotide phosphate (NADPH) oxidase activity, increasing LPO, and impairing mitochondrial redox homeostasis within ovarian and adipose tissues (73). These redox disturbances not only compromise oocyte quality and follicular maturation but also intensify local inflammatory signaling via NF-κB activation. Chronic inflammation, another hallmark of PCOS, arises from ROS-mediated activation of redox-sensitive transcription factors, which upregulate proinflammatory cytokines such as TNF-α, IL-6, and CRP (32, 71). These mediators aggravate insulin signaling defects and sustain androgen biosynthesis, forming a vicious triad of OS, endocrine dysfunction, and inflammation. The convergence of these processes underscores that OS is not an epiphenomenon but a mechanistic nexus connecting endocrine and metabolic abnormalities in PCOS. Importantly, redox imbalance acts as the molecular bridge through which IR and HA reinforce each other while maintaining a chronic inflammatory state (7, 37). Addressing this triad requires interventions capable of re-establishing redox equilibrium alongside hormonal and metabolic restoration, an emerging rationale for exploring antioxidant-endocrine modulators such as vitamin D, MI, and melatonin as integrated therapeutic adjuncts in PCOS management.

### 2.4 Redox-endocrine vicious cycle in PCOS

The pathophysiology of PCOS is underlined by a self-reinforcing redox-endocrine loop that integrates OS, hormonal dysregulation, and metabolic dysfunction into a persistent pathological circuit. Excessive generation of ROS within ovarian, adipose, and hepatic tissues overpowers antioxidant defenses, leading to oxidative damage of lipids, proteins, and mitochondrial DNA (74). This oxidative burden impairs insulin receptor signaling through serine phosphorylation of IRS, fostering systemic insulin resistance and compensatory hyperinsulinemia (75). Elevated insulin levels, in turn, amplify ovarian theca-cell steroidogenesis via upregulation of CYP17A1 and synergistic stimulation by luteinizing hormone, culminating in hyperandrogenemia (76).

**Figure 1:**
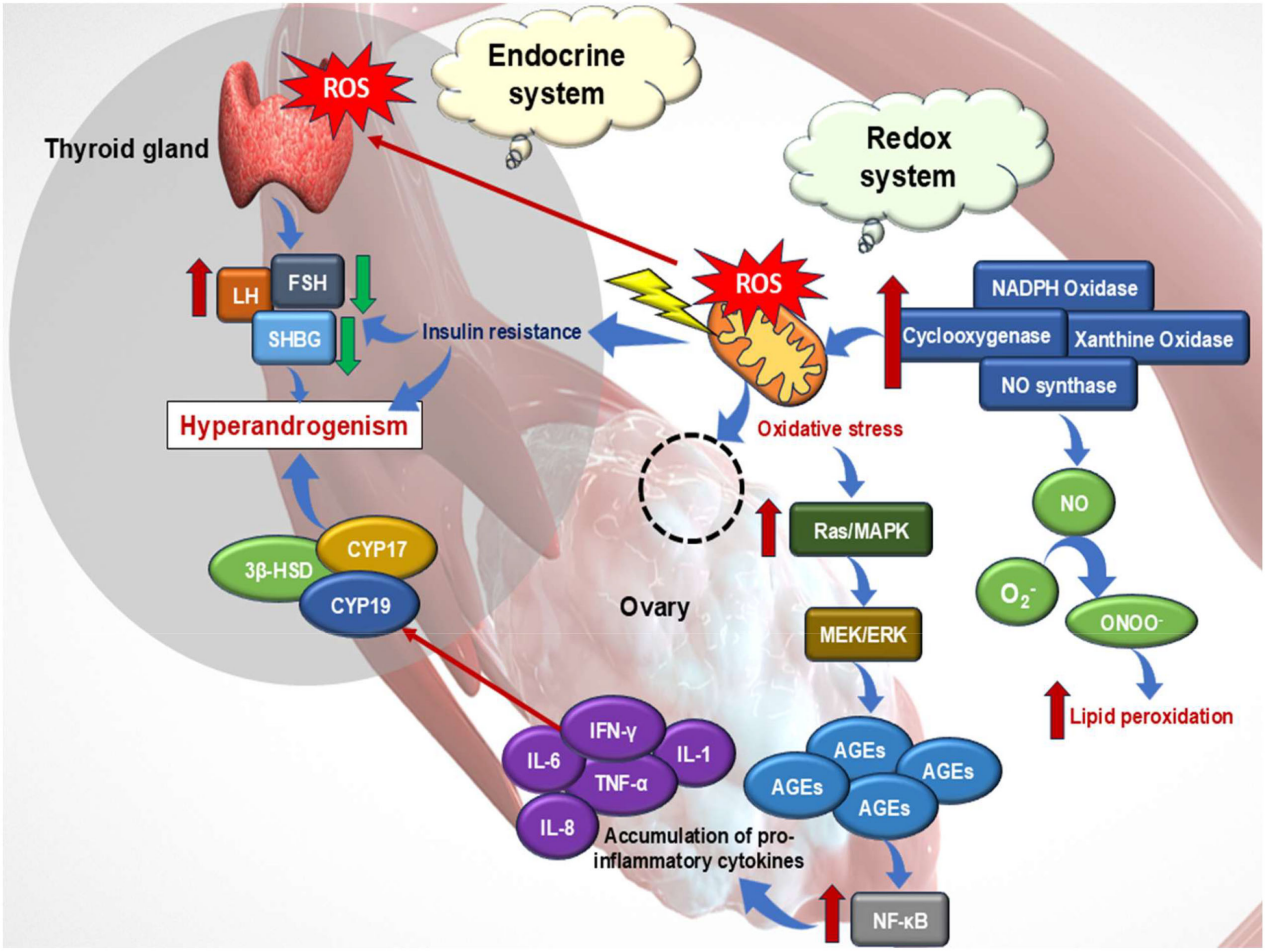
Schematic representation of the involvement of redox imbalance and endocrine dysregulation in the ovary, leading to hyperandrogenism or polycystic ovary syndrome (PCOS). Enzymes such as nicotinamide adenine dinucleotide phosphate (NADPH) oxidase, nitric oxide synthase, xanthine oxidase, and cyclooxygenase increase the reactive oxygen species (ROS) generation, which further induces lipid peroxidation and oxidative stress. This increased ROS level alters the balance of sex hormone-binding globulin (SHBG), luteinizing hormone (LH), follicle-stimulating hormone (FSH), and insulin, triggering insulin resistance. These factors primarily contribute to hyperandrogenism. Additionally, upregulated steroidogenic enzymes, such as 3β-hydroxysteroid dehydrogenase (3β-HSD), CYP17, and CYP19, further amplify hyperandrogenism. Activation of intracellular signaling cascades, such as MEK/ERK and Ras/MAPK, leads to increased NF-κB activation and accumulation of advanced glycation end products (AGEs). These oxidative stress pathways lead to the accumulation of pro-inflammatory cytokines (IL-1, IL-6, IL-8, TNF-α, and IFN-γ), thereby driving ovarian inflammation.

Androgen excess exacerbates redox disequilibrium by promoting NADPH oxidase activity and mitochondrial ROS production, while simultaneously impairing follicular maturation and oocyte quality (55). The resulting follicular arrest perpetuates chronic anovulation and hyperandrogenic feedback on the hypothalamic-pituitary-ovarian (HPO) axis (76, 77). Parallel to these endocrine perturbations, IR drives lipotoxicity and systemic inflammation, characterized by increased TNF-α, IL-6, and C-reactive protein, which further suppresses insulin sensitivity and antioxidant enzyme expression (78). This reciprocal amplification between OS and hormonal imbalance forms a pathological loop that sustains the metabolic-reproductive continuum of PCOS. Moreover, chronic low-grade inflammation acts as a molecular bridge, linking redox stress to endocrine dysfunction. NF-κB activation and impaired Nrf2 signaling suppress mitochondrial biogenesis and redox recovery, strengthening the persistence of the cycle (71). Breaking this vicious network demands simultaneous targeting of OS, mitochondrial dysfunction, and hormonal imbalance. Thus, agents capable of restoring redox equilibrium while recalibrating endocrine signaling—such as vitamin D, myo-inositol, and melatonin—represent mechanistically coherent candidates to interrupt this loop and reestablish physiological homeostasis in PCOS. **Figure 1** represents the involvement of redox imbalance and endocrine dysregulation in the ovary, leading to hyperandrogenism or PCOS.

## 3 Vitamin D: beyond bone health to redox-endocrine modulation

### 3.1 Vitamin D signaling pathways in ovarian and metabolic tissues

**Figure 2:**
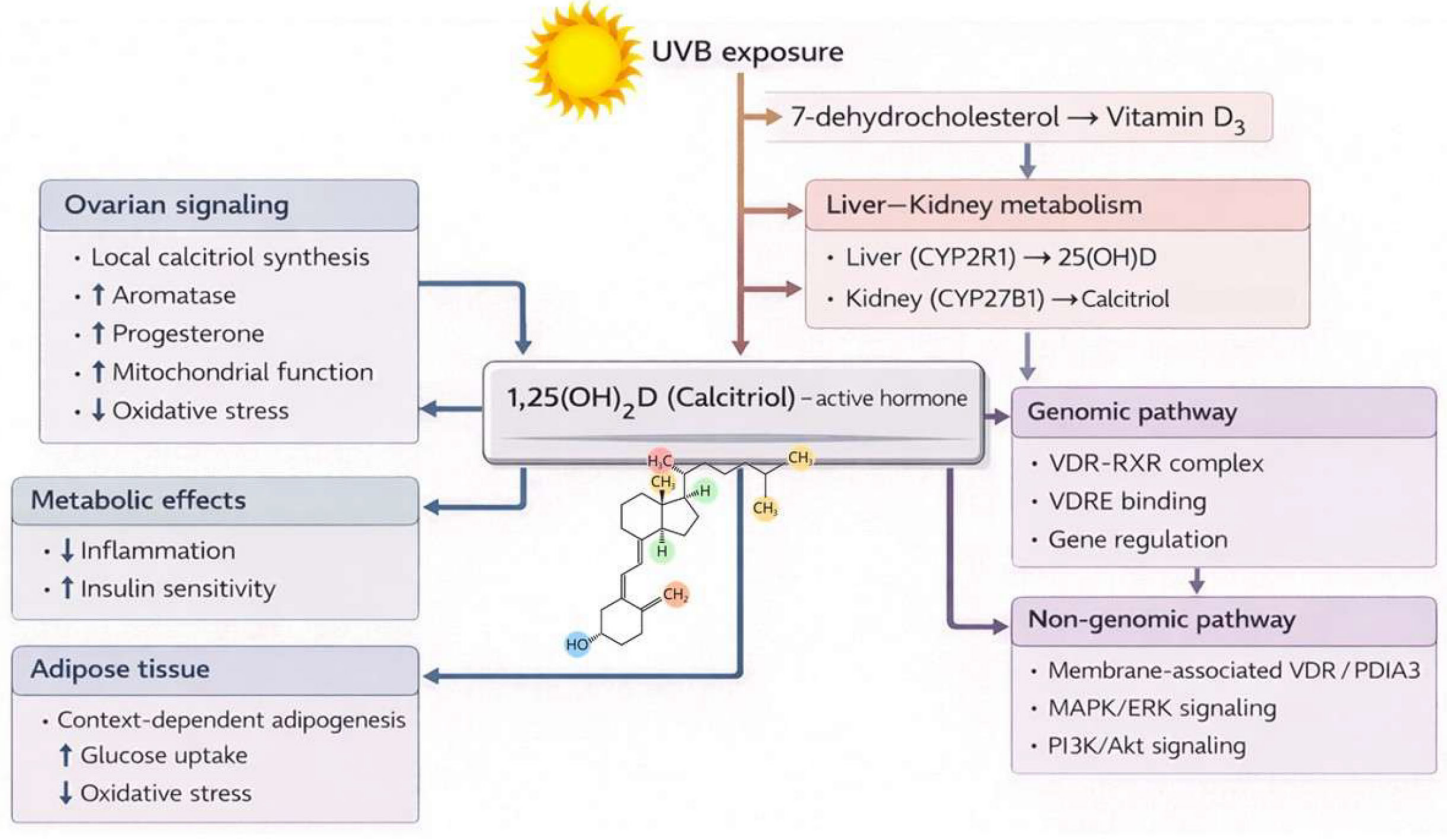
Overview of vitamin D signaling pathways in ovarian and metabolic tissues. Vitamin D_3_ is sequentially hydroxylated to 25(OH)D and the active metabolite 1,25(OH)_2_D (calcitriol) by CYP2R1 and CYP27B1 in hepatic, renal, and extra-renal tissues. Calcitriol signals through genomic pathways via VDR-mediated transcriptional regulation and through rapid non-genomic pathways involving membrane-associated VDR and PDIA3, activating MAPK/ERK and PI3K/Akt signaling. In metabolic tissues, these pathways collectively regulate inflammation, insulin sensitivity, adipogenesis, and oxidative stress. In the ovary, local calcitriol synthesis in granulosa cells supports folliculogenesis, steroidogenesis, mitochondrial function, and redox homeostasis.

Vitamin D, a steroid hormone, is popular for its classical role in calcium and phosphorus homeostasis (79). However, as a pleiotropic regulator of systemic physiology, it exerts its influence in various metabolic tissues by complex signaling pathways. The active metabolite, 1,25-dihydroxyvitamin D3 (calcitriol), is generated via sequential hydroxylations in the liver (by CYP2R1) and kidneys (by CYP27B1) (80). This activation also occurs in extra-renal tissues, such as the ovary and adipose tissue, which express these enzymes, enabling local calcitriol production and establishing tissue-specific responses (81, 82). **Figure 2** provides an integrated schematic overview of vitamin D metabolism and its genomic and non-genomic signaling pathways across metabolic and ovarian tissues, summarizing the mechanisms described in this section. Calcitriol primarily functions by binding to the vitamin D receptor (VDR), a ligand-activated transcription factor. The ligand-bound VDR heterodimerizes with the Retinoid X Receptor (RXR), and this complex recruits co-regulatory proteins to Vitamin D Response Elements (VDREs) in target genes (83). This genomic pathway acts downstream to activate or repress target genes (84). For instance, in the osteoblasts, it activates genes for bone matrix formation (e.g., osteocalcin, osteopontin) and bone remodeling (RANKL) (85). Beyond this, vitamin D also signals via rapid, non-genomic pathways (86). These are mediated by membrane-associated receptors,

VDR itself, or PDIA3, triggering intracellular cascades such as MAPK/ERK and PI3K/Akt. These pathways may converge on genomic responses by phosphorylating VDR or its coactivators (86, 87). On the other hand, VDR also directly interacts with cytosolic signaling molecules, such as inhibiting IκB kinase to suppress the NF-κB pathway, emphasizing its potent anti-inflammatory role (88).

In metabolic tissues, these pathways integrate to maintain homeostasis. In the liver, VDR evolutionarily behaves as a nutrient sensor, wherein its impairment mimics a starvation-like state, leading to an increase in adiposity without diet changes (89). While hepatocytes have low VDR, non-parenchymal Kupffer cells exhibit high expression, where VDR activation curbs inflammation and enhances insulin sensitivity (90). In adipocytes, vitamin D exerts dual, context-dependent effects. It can inhibit adipogenesis in murine preadipocytes by interfering with the Wnt/β-catenin pathway, while promoting it in human mesenchymal stem cells by diverting cell fate from osteogenesis (91). It also enhances glucose uptake via an insulin-independent SIRT1/AMPK/IRS1/GLUT4 axis and mitigates OS through the NOX4/Nrf2 pathway (92). Such an impact on insulin sensitivity in hepatocytes and adipocytes suggests a strong clinical role in type 2 diabetes mellitus (93).

The ovary represents a classic example of localized vitamin D signaling. Granulosa cells of developing follicles express VDR and the enzymes for local calcitriol synthesis, creating an autocrine/paracrine loop (94). This intra-ovarian system is vital for folliculogenesis and steroidogenesis (95). Vitamin D upregulates aromatase (CYP19A1), boosting estrogen production, and modulates progesterone synthesis (96). Emerging evidence highlights its role in enhancing mitochondrial biogenesis and function in granulosa cells, ensuring adequate energy production and reducing OS (97), a role it also plays in skeletal muscle (98). Consequently, vitamin D deficiency is strongly implicated in ovarian pathologies like PCOS and diminished ovarian reserve (99).

### 3.2 Antioxidant and anti-inflammatory roles of vitamin D in PCOS

PCOS has been characterized by a well-documented bidirectional relationship with vitamin D status. This is evidenced by the significant deficiency of its active form, calcitriol, in affected women (94). Beyond its classical role in calcium homeostasis, vitamin D exerts an influence on steroidogenesis (95), inflammation (100), and OS (101), positioning it as a critical modulator in the PCOS phenotype.

Vitamin D influences steroidogenesis by upregulating aromatase gene expression, thereby enhancing the conversion of androgens to estrogens (estradiol and estrone) and stimulating progesterone production (96). This is critically important in PCOS, a state of HA and arrest folliculogenesis. Supporting this, VDR knockout models exhibit impaired folliculogenesis and anovulation, suggesting the significant role of vitamin D in modulating follicular maturation and luteinization (102). This may be attributed to local inflammatory and immune responses (103). Clinically, vitamin D supplementation has been shown to reduce elevated anti-Müllerian hormone (AMH) levels (104), further indicating a restoration of ovarian follicular dynamics.

The therapeutic potential of vitamin D in PCOS is largely attributed to its systemic antioxidant and anti-inflammatory properties (101). A consistent finding is that supplementation significantly reduces high-sensitivity C-reactive protein (hs-CRP), a key marker of systemic inflammation (105). At the cellular level, this anti-inflammatory effect is mediated by downregulating the pro-inflammatory nuclear factor-kappa B (NF-κB) pathway, achieved by decreasing levels of its active, phosphorylated form (pNF-κB) (106). This repression extends to the production of pro-inflammatory cytokines, including TNF-α, IL-1, IFN-γ, and IL-6 (106). Concurrently, vitamin D resists OS, a key driver of PCOS pathology (7). It significantly increases the body’s TAC while reducing MDA, a marker of LPO (101). Vitamin D does not fully normalize OS via MDA in the ovarian tissue in the context of PCOS combined with a high-fat diet (107). It is definitely a modulator in toning down OS. Vitamin D also plays a modulatory role, increasing the expression of critical endogenous antioxidant enzymes, such as SOD1 (108). However, these benefits are specific to certain biomarkers and do not affect all hormones, inflammatory markers (such as NO), or glutathione (101). The influence of vitamin D on glutathione becomes particularly significant when co-supplemented with calcium, as it upregulates glutamate cysteine ligase and glutathione reductase, two key enzymes required for GSH synthesis (109). While vitamin D plays a protective and restorative role in ovarian structure, it should be noted that its anti-inflammatory effects may be limited in advanced or long-standing PCOS (6). These anti-inflammatory and antioxidant mechanisms converge to improve a core feature of PCOS, *i.e.*, IR (88). There are several reasons why vitamin D improves IR, including its direct action on insulin secretion and signaling (93) and its role in regulating calcium as a second messenger for insulin-mediated processes within cells (109). However, one of its driving factors is the reduction of pro-inflammatory cytokines, such as TNF-α, and the increase in pro-inflammatory enzymes (110). While vitamin D supplementation has shown significant evidence in improving PCOS states, for optimal therapeutic impact, evidence suggests a synergistic approach, combining vitamin D with agents like Vitamin D and K (111), calcium (112), omega-3 (113), or probiotics (114), to amplify its restorative potential.

### 3.3 Vitamin D and insulin signaling: effects on glucose homeostasis and HOMA-IR

Vitamin D exerts notable control over insulin signaling and glucose handling (90). Once bound to the nuclear VDR, present in pancreatic β-cells and in adipose tissue, liver, and skeletal muscle, the hormone triggers a series of molecular actions that help maintain insulin sensitivity (88, 98, 99). In particular, vitamin D boosts insulin receptor expression and activates the IRS-1/PI3K/Akt/GLUT4 cascade. The result is more efficient glucose uptake and a measurable improvement in peripheral insulin responsiveness (115). Moreover, vitamin D exerts anti-inflammatory and antioxidant effects, reducing cytokine-mediated interference with insulin signaling and protecting β-cell function (88). These combined actions help restore redox and metabolic balance, thereby interrupting the self-perpetuating redox-endocrine cycle described in PCOS (116). Recent studies have shown that vitamin D deficiency correlates with higher IR and unfavorable HOMA-IR values, while supplementation improves these indices in women with PCOS (117, 118).

Several clinical trials and meta-analyses have assessed the effects of vitamin D supplementation on IR and glucose metabolism in women with PCOS. Most report a significant reduction in fasting insulin levels and HOMA-IR, particularly in women with vitamin D deficiency (119, 120). While most studies show that vitamin D improves insulin sensitivity, the degree of improvement varies. Factors such as dosage, treatment duration, and baseline vitamin D status significantly influence these variations. Women who are vitamin D-deficient or obese tend to show greater improvements in HOMA-IR and fasting glucose compared with those with sufficient levels (117). Genetic variability in the VDR may also affect individual responsiveness to supplementation (121). Furthermore, as mentioned earlier, research combining vitamin D with other insulin-sensitizing substances like calcium, omega-3 fatty acids, or MI frequently shows greater metabolic advantages than vitamin D alone (113).

### 3.4 Clinical evidence of vitamin D supplementation in PCOS: reproductive, metabolic, and OS outcomes

Clinical trials investigating vitamin D supplementation in PCOS report a heterogeneous but progressively clarifying signal across reproductive, metabolic, and oxidative-inflammatory domains (122). On the reproductive axis, recent meta-analyses and RCTs suggest modest improvements in ovulation-induction pregnancy rates and androgen milieu, with reductions in LH and total testosterone in some studies (123); however, effect sizes vary with baseline 25-hydroxyvitamin D [25(OH)D], adiposity, and co-interventions. Higher pregnancy rates have been reported with adjunctive vitamin D during ovulation induction, while several RCTs showed cycle regularization and improved ovulation in subgroups with elevated LH/FSH ratios (124), yet other trials reported null effects on primary reproductive endpoints, underscoring between-study heterogeneity in dose (e.g., daily 1,000-4,000 IU vs intermittent 50,000 IU), duration (8–24 weeks), and concomitant therapy.

Metabolic outcomes are similarly mixed but trend favorably when deficiency is corrected, and IR is pronounced. Several trials and updated analyses indicate improvements in fasting insulin, HOMA-IR, BMI/waist indices, and lipid parameters, particularly among women with obesity or IR and low baseline 25(OH)D, whereas pooled estimates from other RCTs fail to show consistent effects on fasting glucose or HOMA-IR, likely reflecting underpowered studies and short follow-up (118). Collectively, these data support a ‘responsive phenotype ‘: women with 25(OH)D insufficiency and higher IR appear more likely to benefit metabolically. By contrast, OS readouts show a more coherent signal. Multiple meta-analyses of RCTs demonstrate significant reductions in MDA and high-sensitivity C-reactive protein (hs-CRP), accompanied by concurrent increases in TAC, indicating tangible redox and inflammatory mitigation with supplementation. Subgroup analyses suggest that 12-week courses and lower daily doses (≤1,000 IU/day) can be effective for these endpoints, though dose-response relationships remain imprecise. Notably, improvements in TAC/MDA occur even when glycemic indices are unchanged, implying a primary redox effect that may precede metabolic reprogramming. There are two limitations to translating these findings. First, publication bias and small-study effects cannot be excluded, and many trials do not stratify by obesity, phenotype, or baseline 25(OH)D, key modifiers of response. Second, synergy with other agents (e.g., MI) is biologically plausible but clinically under-tested in factorial designs; the extant literature is dominated by inositol-centric trials with limited testing of vitamin D interactions. Overall, the clinical evidence supports vitamin D as an adjunct that reliably improves redox-inflammation biomarkers and may enhance reproductive and metabolic outcomes in selected PCOS subgroups. A precision approach, baseline 25(OH)D assessment, IR/obesity stratification, and standardized dosing/duration should anchor future trials and practice.

Clinical evidence on vitamin D in PCOS remains inconsistent because trials differ widely in baseline status, dosing, co-interventions, and outcome definitions. Key preclinical and clinical studies evaluating vitamin D supplementation in PCOS are summarized in **Table 1**. Small samples, brief follow-up, and variable assays add further noise. These limitations obscure actual effects and highlight the need for rigorously designed phenotype-stratified, repletion-verified studies.

**Table 1:** Preclinical and clinical studies of vitamin D to target PCOS.

Type of study	Study objectives	Notable outcomes	References
Preclinical	This study assessed the synergistic effects of vitamin D and MitoQ10 in a DHEA-induced mouse model.	This treatment significantly reduced levels of MDA, SOD, LH/FSH, progesterone, and estradiol. Histological observations showed the development of corpora lutea, antral follicles, and atretic follicles.	(125)
Preclinical	To investigate the effects of 120ng/100g/week vitamin D treatment in a 6 mg/kg/day DHEA-treated rat model for a period of 28 days.	Light microscopic techniques revealed the formation of newer cystic and atretic follicles. Electron microscopy revealed lipid accumulation in interstitial cells, thickening of theca cell layers, and attenuation of the granulosa cell layers in cystic follicles.	(126)
Preclinical	This study elucidated the effects of vitamin D3 on steroidogenesis and the AMPK pathway in granulosa cells obtained from DHEA-treated PCOS mice model.	Immunoblotting revealed that vitamin D3 treatment accelerated the phosphorylation of acetyl-CoA carboxylase and AMPKα. This treatment significantly reduced gene expression of steroidogenic enzymes (3β-HSD, Cyp19α1, StAR, Cyp11α1, and P450scc). Radioimmunoassay revealed reduced production of 17β-estradiol and progesterone.	(127)
Preclinical	This research revealed the effects of a low (1.3 µg/kg/week) dose of vitamin D in a DHEA-induced PCOS and a high-fat diet-induced obese mouse model for 40 days.	Treatment with the low dose of vitamin D significantly reduced ovarian and liver weight and testosterone levels, while improving total cholesterol levels compared with the disease group.	(128)
Preclinical	This research aimed to study the pharmacological actions of vitamin D3 on mitochondrial biogenesis in granulosa cells in a PCOS-induced rodent model.	The results showed that vitamin D3 modulated the MAPK-ERK1/2 pathway to reduce the mitochondrial membrane potential and ROS levels. The upregulation of anti-apoptotic genes (B-cell lymphoma-2), antioxidant enzymes (CAT, GPx, SOD), and mitochondrial biogenesis factors (NRF and PPARγ) indicated the reversal of PCOS effects.	(129)
Clinical	A controlled, randomized, and double-blinded study to investigate the synergistic effects of clomiphene citrate and vitamin D (6000 IU for 8 weeks followed by 2000 IU till pregnancy) supplementation in female subjects with PCOS.	This treatment significantly increased the number of mature follicles and normalized the pregnancy rate in female subjects treated with vitamin D and clomiphene compared to the control group.	(130)
Clinical	A quadruple masked, parallel assigned, and randomized treatment to study the effects of regulatory peptides (follistatin and adipokines) and vitamin D in PCOS-affected subjects.	Vitamin D supplementation significantly improved cumulative clomiphene-induced ovulatory rates, as assessed by ultrasound folliculometry, for 6 months. A significant increase in the number of follicles with a diameter of >18 mm was observed. The fetal heart rate was normalized in the vitamin D-treated group as observed through transvaginal ultrasound.	(131)
Clinical	A comparative study was conducted to elucidate the effects of vitamin D (5,000 IU) and metformin (1000 mg) in an open-label randomized clinical trial in female subjects with PCOS, mild depression, insulin resistance, and vitamin D insufficiency.	The treatment significantly improved serum vitamin D levels, Beck Depression Inventory depression score, and State-Trait Anxiety Inventory Score, and reduced fasting blood glucose levels and insulin resistance compared with the control treatment.	(132)
Clinical	This study investigated the combined effects of clomiphene (100 mg), metformin (2000 mg), and vitamin D (100000 IU) in 20-35-year-old females with secondary infertility and PCOS.	This treatment significantly improved follicle growth and maturation at 48 h, the 3rd month, and the 5th month, as observed by transvaginal ultrasound. The treatment also improved the pregnancy outcomes compared to the control group.	(133)
Clinical	To study the effects of elemental calcium (1000 mg), vitamin D2 (50000 IU), vitamin D3 (2000 IU), and medroxyprogesterone (10 mg) in obese premenopausal subjects with PCOS.	The results showed a significant reduction in serum HbA1c, insulin resistance, fasting glucose, and C-reactive protein in the treatment group compared with the control group.	(134)

DHEA, dehydroepiandrosterone; MDA, malondialdehyde; SOD, superoxide dismutase; LH, leutinizing hormone; FSH, follicle stimulating hormone; PCOS, polycystic ovary syndrome; CoA, coenzyme A; AMPKα, adenosine monophosphate-activated protein kinase alpha; 3β-HSD, 3-beta-hydroxysteroid dehydrogenase; Cyp, cytochrome; MAPK-ERK1/2, mitogen-activated protein kinase, extracellular signal-regulated kinase ½; ROS, reactive oxygen species; CAT, catalase; GPx, glutathione perioxidase; NRF, nuclear related factor; PPARγ, peroxisome proliferator-activated receptor gamma; HbA1c, glycated hemoglobin.

## 4 Myo-inositol: insulin sensitizer and redox regulator

### 4.1 Biochemistry of MI and inositolphosphoglycans in insulin signaling

MI is a central molecule in the biochemistry of insulin signaling; it acts both as a free intracellular compound and as the structural foundation for derivatives such as phosphatidylinositols and inositolphosphoglycans (IPGs) (135, 136). In insulin-responsive tissues, the equilibrium between MI and its epimer D-chiro-inositol (DCI) is important because insulin promotes the epimerase-mediated conversion of MI into DCI, which is then incorporated into GPI-anchored proteins and released as DCI-IPGs following insulin stimulation (137). These IPGs serve as second messengers, facilitating downstream activation of IRS, PI3K/Akt pathways, and ultimately promoting glucose uptake and metabolism (135).

IR, a hallmark of PCOS, is characterized by a reduction in the effectiveness of insulin transduction and impaired epimerase activity, which limits the release of DCI from muscle and adipose tissue (138). This restriction impedes proper glucose utilization, aggravating metabolic disruptions associated with PCOS (139). Ovarian tissue retains insulin sensitivity even during systemic IR, leading to an excessive local conversion of MI to DCI (140). This ‘inositol paradox’ causes a marked reduction in the MI/DCI ratio in ovarian follicular fluid compared to healthy controls (0.2:1 in PCOS patients vs. 100:1 in healthy women) (141). High DCI levels in the ovary can further exacerbate androgen production and impair aromatase expression, aggravating the hormonal imbalances seen in PCOS. Conversely, MI modulates insulin effects and enhances aromatase and FSH receptor expression, contributing to improved ovarian hormone synthesis (142).

Clinical therapy research demonstrates that supplementation with MI alone or in combination with DCI in a physiological ratio (typically 40:1) improves insulin sensitivity, lowers hyperinsulinemia, and enhances reproductive outcomes in PCOS patients, even in the absence of overt IR (140). Inositol-based treatments are gaining recognition for their safety, efficacy, and favorable side effect profile compared to conventional pharmacological therapies. These benefits stem not only from improved insulin action but also from direct effects on ovarian steroidogenesis, metabolic regulation, and cellular signaling pathways central to glucose homeostasis and reproductive function (143). The role of MI in the pathophysiology of PCOS highlights its significance in both metabolic and reproductive health, validating its status as a promising adjunct or alternative in clinical management (141).

### 4.2 MI and ovarian function: oocyte maturation, follicular fluid microenvironment, and FSH signaling

MI plays a pivotal role in ovarian physiology, exerting effects on oocyte maturation, modulation of the follicular fluid microenvironment, and enhancement of follicle-stimulating hormone (FSH) signaling. As a crucial second messenger within granulosa cells, MI is central to transducing FSH signals, favoring the selection of the dominant follicle, and supporting oocyte development throughout oogenesis. There is consistent demonstration that MI supplementation is associated with significant improvements in oocyte quality and fertilization rates, especially among women with PCOS and those undergoing assisted reproductive technology (ART) (144). A recent meta-analysis found that MI administration increased the proportion of mature metaphase II (MII) oocytes retrieved, with pronounced benefits seen in PCOS and non-obese PCOS populations compared to controls. However, the impact on women with poor ovarian response was less marked (145).

The action of MI is not limited to its effect on oocyte maturation. Its concentration in follicular fluid is considered a biomarker of follicle and oocyte quality. High levels of MI in follicular fluid are directly correlated with improved oocyte competence, more favorable embryo development, and higher fertilization rates (136). These redox-regulatory effects are particularly relevant in PCOS, where a disrupted follicular microenvironment and heightened OS negatively impact fertility outcomes (146), and are discussed in greater mechanistic detail in Section 4.3.

**Figure 3:**
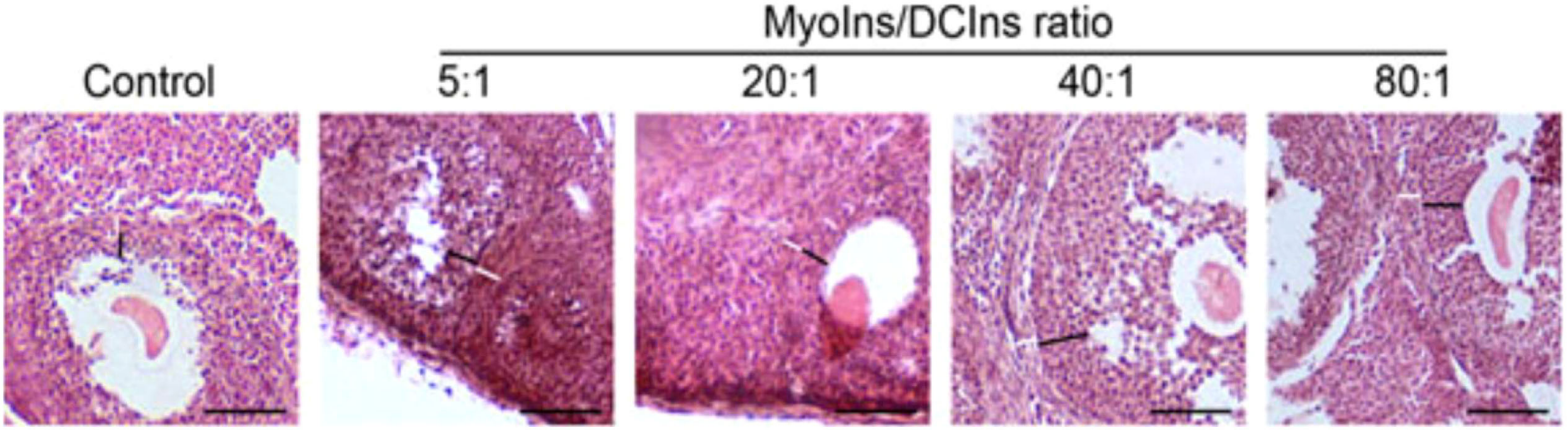
Thickness of theca and granulosa cell layers in ovarian follicles from mice after ten‐day natural recovery (control) or treatments with myo-inositol (MyoIns) and D-chiro-inositol (DCIns). (5:1, 20:1, 40:1 or 80:1). The thickness of granulosa (black lines) and theca cell layers (white line) changes depending on the administration of various MyoIns and DCIns formulations (Reproduced with permission from Bevilacqua et al., 2019 (150)).

In the context of FSH signaling, MI amplifies granulosa cell responsiveness, facilitating follicular growth, estradiol synthesis, and ovulation. Supporting research suggests that MI supplementation can optimize FSH sensitivity, which is central to improved oocyte maturation and folliculogenesis (147). Notably, supplementation may reduce the required dose and duration of exogenous gonadotropins during controlled ovarian stimulation and is linked to higher pregnancy rates in ART cycles (148), primarily when used in combination with D-chiro-inositol at a physiological 40:1 ratio (149). Experimental evidence supports the importance of the MI/DCI ratio in ovarian physiology, as different formulations induce distinct changes in granulosa and theca cell architecture, with the physiological 40:1 ratio preserving follicular structure compared to DCI-enriched conditions (**Figure 3**). There are several studies that support a reduction in the number of degenerated oocytes and better overall embryo quality, suggesting both direct and indirect benefits of MI therapy (151).

### 4.3 Impact on OS, mitochondrial activity, and oocyte quality

MI and its bioactive derivatives, particularly IPGs, have emerged as crucial modulators of insulin signaling, redox homeostasis, and mitochondrial bioenergetics in PCOS (147). Acting as second messengers in the insulin cascade, IPGs facilitate glucose uptake, glycogen synthesis, and lipid metabolism through the activation of key enzymes such as pyruvate dehydrogenase and glycogen synthase (135). In PCOS, where OS and IR intersect, MI supplementation restores downstream insulin sensitivity while attenuating ROS generation, a dual action that integrates metabolic correction with redox stabilization (27).

Experimental and clinical studies collectively reveal that MI modulates cellular redox balance by enhancing antioxidant enzyme activities and reducing oxidative biomarkers such as malondialdehyde (MDA) and nitric oxide derivatives (146, 149, 151). By improving mitochondrial membrane potential and ATP synthesis, MI protects granulosa and cumulus cells from oxidative injury, thereby preserving oocyte competence (152). *In vitro* evidence demonstrates that MI enhances mitochondrial biogenesis and normalizes mitochondrial ultrastructure in oocytes from PCOS animal models, counteracting ROS-mediated mitochondrial depolarization and DNA damage. Moreover, by stabilizing mitochondrial function, MI contributes to the maintenance of cytoplasmic maturation processes essential for proper meiotic spindle assembly and chromosomal segregation, mechanistic correlates of improved oocyte quality and fertilization potential (153).

At the tissue level, MI supplementation is associated with reduced oxidative load in the follicular environment and increased activity of antioxidant defenses such as GPx and SOD, changes that align with improved follicular integrity and reduced atresia (154, 155). The involvement of IPGs further refines this redox-endocrine dialogue: IPGs act as insulin mimetics, reducing hyperinsulinemia-induced ROS production and limiting LPO in ovarian tissue (135). Nevertheless, heterogeneity in formulations, dosing (typically 2–4 g/day), and treatment duration across studies tempers definitive conclusions. Despite these variations, the convergence of biochemical and clinical data supports a pivotal role of MI and its IPG mediators in reinforcing mitochondrial functions and mitigating OS-driven follicular dysfunction in PCOS. Their mechanistic alignment with both endocrine and redox axes positions MI as a cornerstone in integrative strategies targeting the redox-endocrine-metabolic triad central to PCOS pathophysiology.

### 4.4 Clinical trials: reproductive and metabolic outcomes of MI in PCOS

Across randomized trials and meta-analyses, MI consistently signals benefit for reproductive endpoints in PCOS, though the magnitude varies with phenotype, baseline IR, and treatment context (21, 143, 148). In ovulation-induction and ART settings, MI, alone or as discussed previously, in physiologic combination with D-chiro-inositol (typically 40:1), has been associated with higher clinical pregnancy rates, improved oocyte/embryo quality, and lower gonadotropin requirements (23). Effects appear more pronounced in insulin-resistant or overweight cohorts and in IVF cycles with prior suboptimal response, suggesting a ‘responsive’ subgroup rather than a universal effect (154). Notably, several syntheses now report increased clinical pregnancy with MI ± DCI versus placebo or standard care; small RCTs also document better fertilization rates and embryo morphology, albeit with variable reporting of live birth and moderate heterogeneity (156).

Metabolic outcomes map closely to the role of MI as an insulin-sensitizing second-messenger precursor. Multiple RCTs indicate reductions in fasting insulin and HOMA-IR, with parallel improvements in menstrual regularity and biochemical HA (157). Head-to-head comparisons suggest MI performs similarly to metformin on IR and androgen indices while being better tolerated (158). Some trials even report greater HOMA-IR improvement with MI than metformin, though results are not uniform and follow-up is often short (8–24 weeks). Weight and lipid effects tend to be modest and contingent on baseline adiposity and lifestyle co-interventions (159). Overall, convergent evidence supports MI as a viable first-line adjunct for metabolic-reproductive coupling in PCOS, particularly where metformin intolerance limits adherence. However, interpretation remains cautious, as reproductive endpoints (ongoing pregnancy, live birth) are inconsistently reported and dosing (commonly 2–4 g/day), formulations (MI alone vs 40:1 MI: DCI), co-therapies, and trial quality vary widely, prompting calls for larger, standardized, phenotype-stratified studies. These limitations underscore the need for larger, standardized, phenotype-stratified trials with harmonized reproductive and metabolic endpoints.

### 4.5 Emerging evidence on D-chiro-inositol and combined formulations

Early randomized trials positioned d-chiro-inositol (DCI) as an insulin-sensitizing agent with downstream reproductive benefits (160). In obese women with PCOS, daily DCI improved insulin action, increased ovulation frequency, and lowered androgens and triglycerides versus placebo, signaling a plausible path from metabolic correction to reproductive gain (161). Subsequent work in lean PCOS extended these metabolic and androgenic improvements, although sample sizes were modest and follow-up was short. As the field matured, concern emerged that ovarian epimerase overactivity in PCOS shifts local MI to DCI, creating an ‘ovarian paradox’; systemic IR coexists with intraovarian MI depletion (162). This imbalance provides a mechanistic explanation for why excessive DCI exposure may compromise oocyte competence by further reducing MI-dependent signaling.

This conceptual framework led to the hypothesis, now influential in clinical practice, that restoring a physiologic MI: DCI balance may outperform DCI monotherapy for reproductive endpoints (161). Combination formulations have since proliferated (as already stated, most commonly the 40:1 MI: DCI ratio), reflecting estimated follicular fluid proportions (163). Across RCTs and syntheses, MI ± DCI improves ovulation, menstrual regularity, and surrogate ART outcomes (oocyte/embryo quality), with the 40:1 blend repeatedly associated with favorable endocrine and insulin-resistance profiles and lower gonadotropin requirements (161, 163).

Some comparative and observational studies suggest that combined MI: DCI formulations may confer greater metabolic benefit than MI alone in overweight PCOS populations; however, confidence in these findings is limited by heterogeneity, open-label designs, and short treatment durations. Live-birth data remain sparse (164). Guidelines and recent meta-analyses note the biological promise but still rate the inositol evidence as low-to-moderate because dosing, phenotyping, and endpoints vary widely. Accordingly, while MI: DCI (40:1) appears more physiological for reproductive indications, DCI-leaning regimens may be better suited to metabolic priorities, underscoring the need for phenotype-driven therapeutic strategies (161, 163, 164). Representative preclinical and clinical studies of myo-inositol-based interventions in PCOS are summarized in **Table 2**.

**Table 2:** Preclinical and clinical studies of myo-inositol to target PCOS.

Type of study	Study objectives	Notable outcomes	References
Preclinical	A comparative study between metformin and myo-inositol for their pharmacological effects in improving glucose tolerance and reproductive performance in the letrozole-induced rodent model with PCOS.	Treatment with myo-inositol resulted in similar reductions in body weight, blood glucose, insulin, testosterone, and LH levels compared with the metformin-treated group. Concurrently, the myo-inositol-treated group displayed blood estradiol, FSH, and progesterone levels similar to those of the metformin-treated group.	(165)
Preclinical	To elucidate the molecular mechanism of myo-inositol to target insulin resistance in a rodent model induced with PCOS.	Myo-inositol supplementation significantly downregulated miR-155 and miR-21, p-STAT3, and IL-6 and upregulated the GLUT4 and PPARγ. The result suggested that myo-inositol targeted phospho-STAT3/IL-6 to reduce insulin resistance in PCOS.	(166)
Preclinical	This study aimed to assess the modulation of cytokines and the antioxidant potential of herbal extracts (*W. somnifera* and *U. dioica*), myo-inositol, and probiotics in a PCOS-induced rat model.	Treatment with myo-inositol significantly upregulated the CAT, SOD, and GPx levels in the liver and serum. Additionally, this treatment increased anti-inflammatory cytokine (IL-10) levels and reduced pro-inflammatory cytokines (TNFα and IL-17).	(167)
Preclinical	The synergistic effects of folic acid and myo-inositol for improving the oocyte quality and ovarian morphology were assessed in a PCOS-induced mouse model.	The results showed that 0.36 mg/g of myo-inositol significantly reduced atretic antral follicle diameter and testosterone levels in the DHEA-treated group. Additionally, the granulosa: theca layer thickness ratio in antral follicles and the corpus luteum count were significantly improved in the myo-inositol-treated group.	(168)
Preclinical	To study the molecular effects of myo-inositol in the metabolic disorders in the PCOS rodent model induced with letrozole.	Myo-inositol treatment displayed concentration-dependent upregulation of GLUT4 levels, lipid profiles, and glucose homeostasis. Plasma glucose levels were reduced by 0.85-fold with myo-inositol treatment.	(169)
Clinical	A single masked, parallel assigned, and randomized study to study the synergistic effects of metformin (1500 mg) and myo-inositol (4 g/day) in female subjects with PCOS.	The synergistic treatment improved conception rate, normalized ovulation rates, and improved menstrual regularity compared to myo-inositol treatment alone.	(170)
Clinical	A double-masked, sequentially assigned, and randomized study to evaluate the pharmacological effects of α-lactalbumin, folic acid, and myo-inositol in PCOS subjects.	The combined effects of the active constituents improved the induction of ovulation within 3 months of treatment, as evaluated by ultrasound.	(171)
Clinical	A double-masked, parallel randomized, and controlled study to evaluate the effects of myo-inositol in PCOS subjects with psychiatric and ovarian disorders.	Treatment with myo-inositol showed improvements in psychiatric parameters, such as the McGill pain, Hamilton anxiety, and depression scales.	(172)
Clinical	A comparative study to evaluate the pharmacological effects of metformin and myo-inositol using a parallel-group and randomized clinical study involving subjects with PCOS.	Myo-inositol improved pregnancy rates and normalized menstrual cycles compared with the metformin-treated group.	(173)
Clinical	A prospective and case-control study to study the effects of glucomannan (4 g/day), D-chiro-inositol (0.25 g/day), and myo-inositol (1.75 g/day) in subjects with PCOS for 3 months.	Treatment with this combination normalized plasma cholesterol, triglycerides, insulin, and glucose levels, as well as antral follicle count and ovarian volumes.	(174)

PCOS, polycystic ovary syndrome; LH, leutinizing hormone; FSH, follicle-stimulating hormone; p-STAT3, phosphorylated signal transducer and activator of transcription 3; IL-6, interleukin-6; GLUT-4, glucose transporter type 4; PPARγ, peroxisome proliferator-activated receptor gamma; CAT, catalase; SOD, superoxide dismutase; GPx, glutathione perioxidase; TNFα, tumor necrosis factor alpha; IL-10, interleukin-10; IL-17, interleukin-17; DHEA,

## 5 Melatonin: chrono-redox hormone in ovarian physiology

### 5.1 Melatonin biosynthesis and ovarian localization

Melatonin, an indoleamine, is synthesized by the pineal gland primarily, but also in many other organs such as skin, retina, and ovaries (175, 176). The regulation of melatonin is done via the body’s circadian rhythm, which is controlled by the suprachiasmatic nucleus (SCN). The retina detects light signals from the environment, and in the presence of light, the SCN represses the synthesis of melatonin through inhibitory signals from the SCN to the paraventricular nucleus (177). While in the absence of light, the inhibition is released, allowing for the synthesis of melatonin as the SCN activity drops, which allows the paraventricular nucleus to start activating the intermediolateral cell column, which in turn activates the superior cervical ganglion and finally postganglionic fibers that directly affect the pineal gland with the help of norepinephrine that binds to β_1_-adrenergic receptors to start melatonin synthesis (178).

Melatonin originates from dietary amino acid L-tryptophan. Its synthesis occurs in two broad sites, the pineal gland and various extra-pineal tissues. Within the pineal gland, located at the roof of the third ventricle, pinealocytes convert tryptophan to serotonin through the sequential actions of tryptophan hydroxylase and aromatic amino acid decarboxylase. Serotonin is then acetylated by serotonin N-acetyltransferase to form N-acetylserotonin, which is finally methylated by hydroxyindole-O-methyltransferase to produce melatonin (179). Beyond the pineal gland, several cell types, including enterochromaffin cells of the gut, retinal photoreceptors, and ovarian granulosa-cumulus cells, also generate melatonin (180–182). After synthesis, it enters the bloodstream and cerebrospinal fluid. The liver metabolizes most circulating melatonin via CYP1A2 (and partly CYP2C19), while cells themselves convert it to AFMK and AMK, metabolites known for potent antioxidant and anti-inflammatory activity (183).

Melatonin also plays an important role in the ovaries, as it protects the ovaries and is synthesized by the ovaries (184). Granulosa cells (both cumulus and granulosa cells) express high amounts of NAT and ASMT that help in the production of melatonin, and also receptors MT1 and MT2, which help in the binding of melatonin (22). In the oocytes, melatonin protects the oocyte from OS and damage (185). The follicular fluid contains higher melatonin concentrations than blood, as melatonin is made within that area by cumulus, granulosa cells, and oocytes as well. High levels of melatonin would be beneficial for the oocyte, as it helps in clearing free radicals, upregulates the antioxidant enzymes and even prevents apoptosis of the oocyte (186).

### 5.2 Potent antioxidant role in follicular fluid, oocytes, and granulosa cells

As mentioned earlier, follicular fluid has higher amounts of melatonin when compared to blood, and this is solely owing to the fact that melatonin can be produced locally in the ovary (187). The levels of melatonin are high in the pre-ovulatory phase, as melatonin can diffuse into the follicles. With the LH surge, there is shedding of the basement membrane and invasion of macrophages and neutrophils, which release a lot of ROS (188). Although free radicals participate in physiological shedding, their overproduction can cause unintended tissue injury. Melatonin counteracts this by scavenging reactive species and disrupting the Fenton reaction sequence before additional cellular damage ensues. Along with that, it also helps to maintain the redox balance within the ovarian microenvironment for the oocyte to mature properly (185). Higher levels of 8-hydroxy-2’-deoxyguanosine (8-OHdG) were found to be present, which is a marker for oxidative DNA damage, and when melatonin was introduced, there was an appreciable decrease in the levels of 8-OHdG, which shows that melatonin is a protective factor against ROS-induced DNA injuries (189). In the oocyte, melatonin has similar activities and its metabolites AFMK, AMK have a cascading effect and maintain the same effects as melatonin and helping in antioxidant activity and helping in meiotic spindle formation (190). The granulosa cells are a significant site of ROS, as they help in steroidogenesis, and to combat this, melatonin plays a key role by lowering ROS and MDA levels (191). It also helps in inhibiting Bax/Bak and caspase-3, which are pro-apoptotic proteins, thereby inhibiting apoptosis while also upregulating the activity of SOD and GPx through the Nrf2/ARE pathways (192). Optimal granulosa cell health is crucial, as it enhances cumulus–oocyte communication through gap junctions, supports effective luteinization, and ensures a stable supply of growth factors and nutrients essential for oocyte maturation (193). Beyond its direct antioxidant and anti-apoptotic actions, melatonin also supports oocyte-granulosa cell communication by preserving the expression of key oocyte-derived growth factors such as BMP15 and GDF9, which are essential for folliculogenesis and oocyte competence. Experimental evidence has shown improved expression of these markers along with enhanced follicular development and oocyte quality following melatonin supplementation, as reported by Arık et al. (194).

### 5.3 Effects on steroidogenesis, insulin sensitivity, and circadian regulation

In the female reproductive system, melatonin is synthesized not only systemically but also locally in the ovary, where it modulates steroidogenesis, oocyte maturation, and luteal function (22). Granulosa and theca cells express melatonin receptors (MT1 and MT2), and their activation fine-tunes the steroidogenic enzymatic cascade (195). Evidence from *in vitro* and *in vivo* studies demonstrates that melatonin downregulates steroidogenic acute regulatory (StAR) protein and 3β-hydroxysteroid dehydrogenase expression under OS conditions, thus restraining excessive androgen synthesis (196), an effect particularly relevant in PCOS, where HA disrupts follicular development. Conversely, melatonin supports physiological estradiol synthesis by preserving aromatase activity and maintaining redox stability within granulosa cells (197).

Beyond reproductive signaling, melatonin modulates insulin sensitivity and glucose metabolism through its influence on circadian regulation of pancreatic and peripheral clocks. Melatonin receptor polymorphisms (notably MTNR1B variants) are linked with impaired insulin secretion and heightened diabetes risk, suggesting receptor-level modulation of insulin homeostasis (198). Experimental supplementation studies in women with PCOS reveal that nocturnal melatonin (2–5 mg) improves fasting glucose, HOMA-IR, and adiponectin levels while reducing androgen concentrations and oxidative biomarkers (199). These effects are partly mediated by suppression of nocturnal cortisol and restoration of the physiological melatonin-insulin rhythm, which is often blunted in PCOS due to circadian misalignment (200). Antioxidant actions of melatonin amplify its endocrine effects; it scavenges ROS directly and enhances SOD and GPx activity within ovarian tissue, thereby improving mitochondrial efficiency and oocyte competence. Circadian entrainment of hypothalamic–pituitary-gonadal (HPG) signaling further supports synchronization of gonadotropin release and follicular maturation, reinforcing its reproductive benefits (201). However, interindividual variation in receptor sensitivity, lifestyle-induced circadian disruption, and inconsistent dosing schedules limit uniformity in clinical trial outcomes. Therefore, melatonin acts as a molecular integrator linking redox equilibrium, steroidogenic control, and metabolic timing. In PCOS, where OS, androgen excess, and IR coalesce, melatonin re-establishes a synchronized endocrine rhythm, offering both mechanistic and therapeutic promise as part of a redox-endocrine restoration strategy.

### 5.4 Clinical evidence: melatonin in ART cycles and PCOS-related infertility

Clinical data on melatonin as an adjunct in assisted reproduction (and specifically in PCOS) converge on a plausible benefit for intermediate outcomes, with more cautious signals for hard endpoints. Across randomized trials and meta-analyses in heterogeneous IVF/ICSI populations, melatonin (typically 2–5 mg nocte for 4–12 weeks or during stimulation) increases the number of mature (MII) oocytes, improves fertilization rates, and often enhances embryo quality; pooled effects on clinical pregnancy are small and sometimes non-significant, limited by sample size and trial quality. These findings align with observational links between higher follicular-fluid melatonin and better oocyte/embryo metrics, supporting a mechanistic redox–mitochondrial pathway.

In PCOS cohorts, small RCTs and controlled studies suggest that melatonin pretreatment can improve oocyte maturation (more MII, fewer GV/MI oocytes), reduce oxidative stress biomarkers, and modestly lower androgen levels. These changes translate into higher chemical or clinical pregnancy rates in some studies but not in others. A double-blind RCT in PCOS (5 mg twice daily, 12 weeks) reported improvements in glycemic indices, adipokines, and androgen profile, consistent with melatonin’s insulin-sensitizing and anti-androgenic actions. These features may indirectly raise reproductive efficiency during ovulation induction or ART. Still, live-birth data remain sparse, and heterogeneity in PCOS phenotype, baseline OS, and stimulation protocols complicates pooled inference.

Recent syntheses (2024-2025) sharpen this picture. Meta-analyses show improved fertilization and embryo quality and a probable rise in clinical pregnancy but emphasize low-to-moderate certainty due to small trials, inconsistent dosing schedules, and variable blinding/allocation concealment. Some reviews note no apparent effect on pregnancy in sensitivity analyses, underscoring the gap between biologic plausibility and durable clinical benefit. Safety profiles are favorable (mild somnolence, rare discontinuation), and cost is low, both of which are attractive for adjunctive use while larger, phenotype-stratified RCTs mature.

Two implications follow. First, melatonin appears most useful where oxidative load is high (e.g., PCOS, diminished ovarian reserve, repeat-poor response), aiming to improve oocyte/embryo competence rather than to replace first-line ovulation-induction strategies. Second, future trials should standardize timing (pre-treatment vs stimulation-only), dosing (e.g., 3–5 mg nocte), and core outcomes (ongoing pregnancy, live birth), and incorporate follicular-fluid redox readouts to confirm target engagement in PCOS. Until such data accrue, melatonin is a reasonable, low-risk adjunct to consider in PCOS-related infertility, particularly for patients prioritizing oocyte quality, while counseling that hard reproductive endpoints are not yet definitively established.

### 5.5 Synergy with other bioactives in reducing OS burden

Melatonin’s pleiotropy, direct radical scavenging, mitochondrial protection, and transcriptional control of antioxidant defenses make it a natural hub for combination strategies targeting the redox-endocrine axis in female reproduction (201). Melatonin stabilizes mitochondria, enhances Nrf2-driven antioxidant defenses, and restrains NF-κB activity. When paired with complementary bioactives, these effects broaden PCOS-linked failures, namely OS, insulin resistance, and hyperandrogenism. Its interaction with MI is most developed: MI improves insulin signaling, while melatonin supports granulosa-cell mitochondria, together improving oocyte competence in early studies. Vitamin D, CoQ10, and NAC add anti-inflammatory, ETC-supporting, and GSH-restoring actions, collectively tightening redox control. Although trials remain small and surrogate-heavy, the biological rationale is strong. Present mechanistic and preliminary clinical signals justify exploring melatonin-anchored ‘bioactive cocktails’ to lower oxidative load and enhance oocyte quality in PCOS. Preclinical and clinical evidence supporting melatonin use in PCOS is summarized in **Table 3**.

**Table 3:** Preclinical and clinical studies of melatonin to target PCOS.

Type of study	Study objectives	Notable outcomes	References
Preclinical	The pharmacological effects of 10 mg/kg of melatonin administered for a period of 5 days in DHEA-treated female NMRI mice were observed using histopathological studies.	Melatonin treatment significantly increased the thickness of the theca and granulosa layers and downregulated the numbers of cystic, pre-antral, primary, and primordial follicles.	(202)
Preclinical	Comparative study between melatonin and metformin to assess their metabolic and reproductive effects in rodents with PCOS induced with testosterone.	Melatonin at a dose of 2 mg/kg significantly reduced body mass index, body weight, intra-abdominal fat, and blood glucose, insulin, testosterone, and C-reactive protein levels compared to the metformin (500 mg/kg)-treated group. This dose of melatonin showed HDL, LDL, VLDL, triglycerides, and total cholesterol levels similar to those observed with metformin. Significant reductions in uterine and ovarian weights were observed following melatonin treatment.	(203)
Preclinical	This study examined the effects of melatonin on oocyte quality *in vitro* isolated from C57BL/6 mice with PCOS.	The results indicated concentration-dependent (10–^5^ to 10^-7^ µg/mL) increase in the number of oocytes from 175 to 353.	(204)
Preclinical	A similar comparative study to evaluate the stereological effects of melatonin and metformin on vaginal, ovarian, and uterine cytology in PCOS-induced BALB/c mice.	Both melatonin and metformin regularized estrus cycles and significantly reduced body weight and testosterone levels, resulting in a significant lowering of the numbers of corpus luteum, Graafian, and primordial follicles. Simultaneously, the length and volume of endometrial vessels were increased.	(205)
Preclinical	To evaluate the synergistic effects of metformin and melatonin on caspase 3, Ki-67 proliferation, and apoptosis of interstitial cells in the PCOS-induced rat model.	Synergistically, these bioactives significantly reduced theca interna cell apoptosis, caspase-3 activity in granulosa cells, and Ki-67 expression in granulosa cells. At the same time, Ki-67 expression in interstitial cells was significantly increased.	(206)
Clinical	To evaluate the synergistic effects of melatonin (3 mg), folic acid (200 µg), and myo-inositol (2000 mg) in subjects with irregular menstrual cycles and PCOS undergoing IVF treatment.	This combination enhanced the implantation rate, pregnancy rate, embryo quality, and number of mature oocytes. It also reduced the risk of ovarian hyperstimulation syndrome and increased endometrial thickness.	(207)
Clinical	An open-label and single-group study to study the effects of melatonin (2 mg/day for 6 months) in subjects with PCOS.	This treatment normalized the free androgen index, androstenedione, triglycerides, LDL, HDL, total cholesterol, insulin, and testosterone levels within 6 months.	(208)
Clinical	A parallel assigned, randomized, and open-label clinical trial to assess the combined effects of metformin (500 mg for 7 days) and melatonin (3 mg) in 18-35-year-old female subjects with insulin resistance and PCOS.	This treatment improved physiological homeostasis by reducing insulin resistance. Secondarily, the patients’ waist circumference and BMI were significantly lower than those of the control group.	(209)
Clinical	A prospective and cohort study in infertile women with PCOS undergoing IVF to study the role of melatonin and the renal RAS.	Sandwich ELISA was performed using the urine collected from the subjects. PCOS-affected individuals showed a lower angiotensin-melatonin-creatinine ratio, suggesting a role for the melatonin-RAS axis in PCOS.	(210)
Clinical	A 6-month pilot study was conducted to determine the effects of melatonin on metabolic and endocrine features in females with PCOS.	Treatment with melatonin resulted in significant reductions in androgen and anti-Mullerian hormone levels. Approximately 95% of the subjects exhibited an improvement in their menstrual cycles.	(211)

DHEA, dehydroepiandrosterone; NMRI, Naval Medical Research Institute; PCOS, polycystic ovary syndrome; HDL, high-density lipoprotein; LDL, low-density lipoprotein; VLDL, very low-density lipoprotein; IVF, *in vitro* fertilization; RAS, renin angiotensin system; ELISA, enzyme-linked immunosorbent assay.

## 6 Redox-endocrine triad: mechanistic intersections of vitamin D, MI, and melatonin

### 6.1 Shared molecular targets: OS reduction, mitochondrial stabilization, and anti-inflammatory signaling

The integral pathophysiology of PCOS is linked to chronic OS, mitochondrial dysfunction, and subclinical inflammation, all of which fuel endocrine and metabolic disturbances (7, 58). Vitamin D, MI, and melatonin, though mechanistically distinct, converge on a set of shared molecular targets that regulate redox homeostasis, mitochondrial protection, and inflammatory resolution (22, 112, 158). This intersection defines a promising biochemical synergy capable of modulating PCOS at its molecular core rather than merely attenuating symptoms.

#### 6.1.1 OS reduction

Each of the three agents exerts multilayered control over the oxidative milieu. Vitamin D, acting through the VDR–NF-κB axis, suppresses ROS formation by downregulating NADPH oxidase isoforms and upregulating antioxidant enzymes such as SOD and GPx (212). In granulosa and adipose cells, VDR activation reduces oxidative-inflammatory cascades, indirectly improving insulin signaling and follicular microenvironment stability (213). MI complements this redox balance by enhancing IRS-PI3K/Akt signaling, which reduces ROS generation secondary to hyperinsulinemia and excessive lipid oxidation. By restoring physiological insulin flux, MI lowers the oxidative burden and restores redox-sensitive transcriptional control (169). Melatonin, distinctively, neutralizes ROS and reactive nitrogen species directly and also upregulates antioxidant genes via Nrf2-Keap1 activation (214). Beyond this, it attenuates mitochondrial ROS generation by optimizing ETC coupling and preventing cardiolipin peroxidation (215). Thus, these molecules reinforce antioxidant defense both transcriptionally and metabolically, closing the feedback loop between IR, OS, and cellular dysfunction in PCOS.

#### 6.1.2 Mitochondrial stabilization

Mitochondrial impairment lies at the center of PCOS-related oocyte dysfunction, apoptosis, and metabolic inflexibility (27). Vitamin D enhances mitochondrial oxidative phosphorylation efficiency by regulating calcium flux and increasing expression of respiratory complex subunits, particularly in oocytes and skeletal muscle (109). MI restores mitochondrial biogenesis indirectly through improved glucose utilization and reduced LPO, contributing to normalized ATP generation (135). Melatonin plays the most direct role; it binds cardiolipin, stabilizes the inner mitochondrial membrane, and prevents cytochrome c leakage, thereby reducing apoptosis and maintaining mitochondrial potential (216). Experimental models show that combined vitamin D and melatonin supplementation restores mitochondrial morphology, decreases mtDNA damage, and improves follicular development (217, 218). The triad, therefore, aligns functionally in sustaining mitochondrial resilience: vitamin D provides genomic modulation, MI ensures metabolic substrate balance, and melatonin executes real-time oxidative shielding and bioenergetic stabilization.

#### 6.1.3 Anti*-*inflammatory and endocrine signaling

Chronic low-grade inflammation acts as both driver and consequence of redox-endocrine imbalance in PCOS (71). Vitamin D downregulates pro-inflammatory cytokines (IL-6, TNF-α, CRP) through inhibition of NF-κB translocation and upregulation of IL-10, a key anti-inflammatory mediator (219). It also suppresses macrophage M1 polarization within adipose tissue, mitigating endocrine crosstalk that sustains androgen excess (220). MI exerts indirect anti-inflammatory actions by normalizing insulin and lipid metabolism, reducing activation of the NLRP3 inflammasome, and decreasing systemic cytokine output (Ref). Melatonin, acting through MT1/MT2 and cytosolic binding sites, modulates both the NF-κB and JAK/STAT pathways, curbing inflammatory gene transcription while preserving glucocorticoid sensitivity (221). Its synchronization of circadian rhythms further restores hormonal timing across the HPO axis, tempering aberrant LH surges and cortisol dysregulation (215).

### 6.2 Targeting the redox-endocrine triad with the beneficial trio

The potential interaction between vitamin D, MI, and melatonin can be understood through their overlapping actions on key molecular pathways, including Nrf2, NF-κB, and PI3K/Akt signaling, as well as mitochondrial redox regulation. Vitamin D contributes primarily through genomic regulation of antioxidants and inflammatory pathways (84). MI supports metabolic balance by improving insulin signaling and substrate utilization (142), while melatonin acts at the mitochondrial level to limit oxidative damage and preserve cellular function (222). Together, these complementary actions suggest a biologically plausible framework in which these agents may influence the redox-endocrine disturbances observed in PCOS.

Some early preclinical and small-scale clinical studies have explored combinations of these compounds and reported improvements in oxidative stress markers, such as increased TAC and reduced MDA, along with better oocyte-related parameters (218, 223). However, these findings should be interpreted with caution. Most available studies are limited by small sample sizes, short durations, and variability in study design, and importantly, they often include additional components alongside the three agents. For example, a non-randomized prospective observational study reported that a multi-component supplement containing vitamin D, folic acid, melatonin, and MI improved insulin resistance, androgen levels, and menstrual regularity over six months in women with PCOS (224). While these findings are encouraging, the absence of randomization and the inclusion of multiple active ingredients make it difficult to attribute the observed effects specifically to the proposed triad. Similarly, a randomized study conducted in women undergoing intracytoplasmic sperm injection (ICSI) evaluated a formulation containing vitamin D3, melatonin, and MI and found improvements in oocyte and blastocyst quality, without a significant effect on overall pregnancy outcomes (218). Another pilot randomized study using a combination that included vitamin D3, MI, melatonin, and folic acid reported improved implantation rates in IVF settings (223). These studies provide preliminary clinical signals, but they are primarily focused on assisted reproduction outcomes and do not isolate the independent contribution of each component.

**Figure 4:**
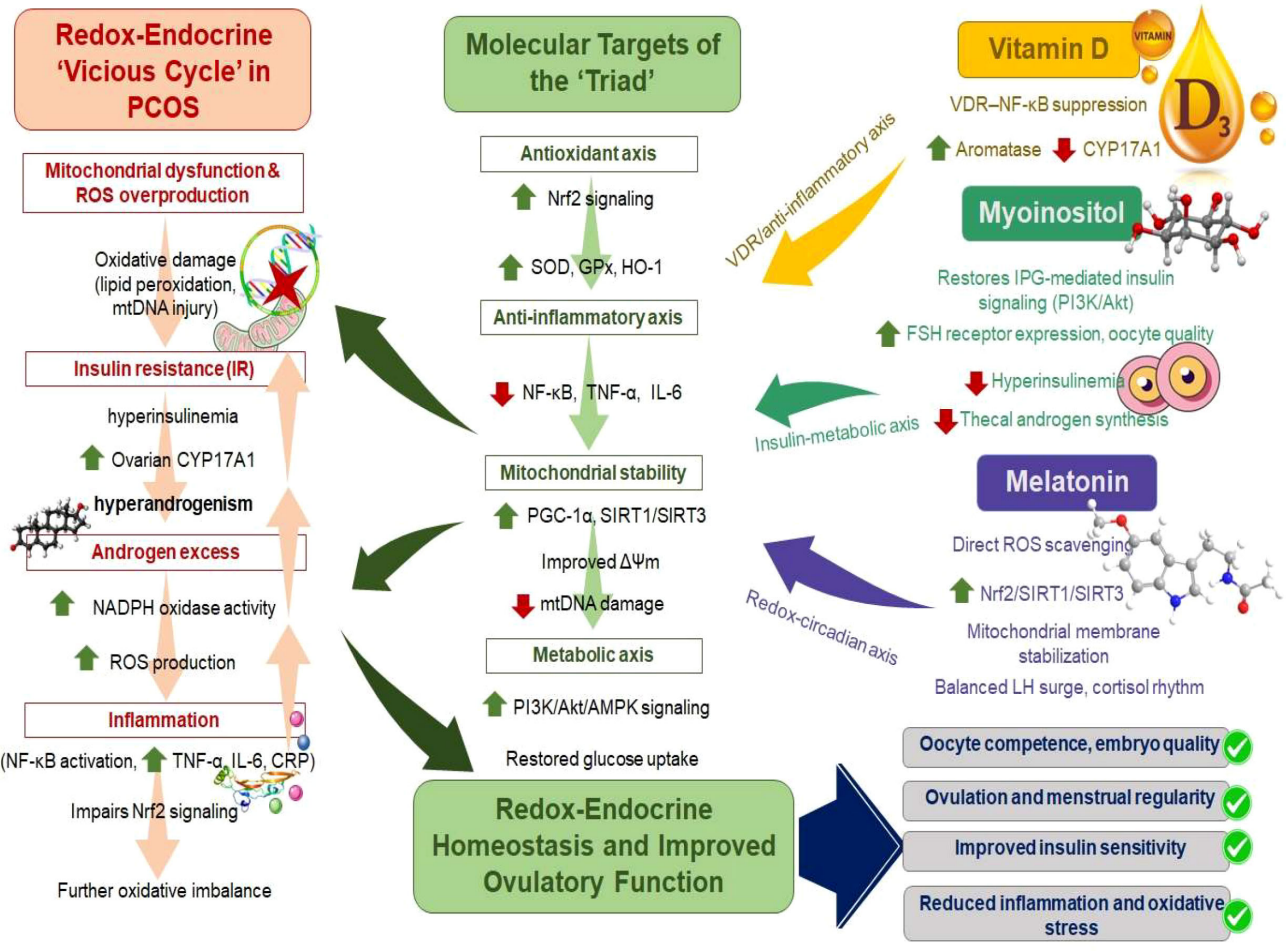
Diagrammatic illustration of the redox-endocrine triad in PCOS and therapeutic intervention with vitamin D, melatonin, and myo-inositol. Mitochondrial dysregulation leads to overproduction of ROS, triggering chronic inflammation, hyperandrogenism, and insulin resistance. Alterations in key metabolic (PI3K/Akt/AMPK), mitochondrial (SIRT1/3 and PGC-1α), anti-inflammatory (NF-κB), and antioxidant (Nrf2) pathways will restore redox-endocrine balance. Vitamin D, myo-inositol, and melatonin synergistically reduce inflammation and oxidative stress, thereby improving oocyte quality, androgen synthesis, and insulin sensitivity, thereby further improving menstrual regularity and ovulation patterns in PCOS.

Thus, the current body of evidence indicate that the combined use of vitamin D, MI, and melatonin is biologically plausible and may provide additive effects at both molecular and cellular levels. Mechanistically, these agents share overlapping and complementary actions that could synergistically target the redox-endocrine disturbances characteristic of PCOS. **Figure 4** illustrates the redox-endocrine triad in PCOS and possible therapeutic intervention with vitamin D, melatonin, and myo-inositol. However, while early studies and preclinical findings are promising, robust clinical data directly supporting the efficacy of this specific combination as a standardized therapeutic strategy remains limited. As such, the proposal to use vitamin D, MI, and melatonin together should currently be regarded as an emerging area of research interest rather than an established or universally recommended treatment option for PCOS. To validate the potential benefits and clarify the therapeutic role of this combination, there is a clear need for well-designed, adequately powered randomized controlled trials. Such studies should utilize standardized formulations and clearly defined clinical endpoints to determine whether this intervention can consistently deliver clinically meaningful improvements in PCOS management.

### 6.3 Complementary endocrine actions: insulin sensitization, androgen modulation, and ovulatory regulation

The clinical and molecular intersections of vitamin D, MI, and melatonin converge on three tightly connected endocrine domains central to the pathophysiology of PCOS: IR, androgen excess, and ovulatory dysfunction. Though each compound acts through distinct biochemical pathways, their overlapping molecular targets reveal an integrative network capable of restoring insulin signaling, modulating steroidogenesis, and re-establishing ovulatory rhythm, reflecting a multidimensional redox-endocrine homeostasis that current pharmacotherapies only partially address.

#### 6.3.1 Insulin sensitization

IR is both a driver and a consequence of OS and HA in PCOS (75). MI serves as a key mediator of insulin signaling by generating inositolphosphoglycan (IPG) second messengers, which facilitate glucose uptake, glycogen synthesis, and lipid oxidation (135). In PCOS, a deficiency in MI-derived IPGs impairs downstream PI3K/Akt activation, producing blunted GLUT4 translocation and chronic hyperinsulinemia (75). Supplementation with MI restores this signaling axis, reducing IR and normalizing compensatory hyperinsulinemia that perpetuates ovarian androgen synthesis (164). Vitamin D complements this mechanism through transcriptional control of insulin receptor gene expression and calcium homeostasis, which fine-tunes insulin vesicle exocytosis (117). The vitamin D receptor (VDR)-PPARγ crosstalk enhances adipocyte insulin responsiveness, while its suppression of the renin-angiotensin system lowers OS that interferes with insulin signaling (225). Melatonin further reinforces insulin sensitivity by synchronizing circadian insulin secretion, improving mitochondrial efficiency in hepatocytes and skeletal muscle, and attenuating the nocturnal cortisol overshoot typical in PCOS (27). Experimental and clinical data demonstrate that nocturnal melatonin supplementation lowers fasting insulin and HOMA-IR, likely through modulation of MT1/MT2 receptor-mediated AMPK activation and redox stabilization of insulin-signaling proteins (226, 227).

#### 6.3.2 Insulin modulation

HA represents the endocrine hallmark of PCOS, sustained mainly by hyperinsulinemia, OS, and disrupted steroidogenic signaling (25). Vitamin D suppresses androgen biosynthesis via downregulation of CYP17A1 and modulation of anti-Müllerian hormone (AMH) expression in granulosa cells, indirectly improving follicular responsiveness to FSH (96). Its deficiency correlates with elevated testosterone and LH/FSH ratios, both of which improve upon repletion. MI, by mitigating IR, decreases insulin-driven thecal cell steroidogenesis and hepatic inhibition of sex hormone-binding globulin (SHBG), effectively reducing free androgen bioavailability (228). Melatonin complements these actions through two interconnected mechanisms, i.e., it restrains OS-induced overactivation of steroidogenic acute regulatory (StAR) protein and 3β-hydroxysteroid dehydrogenase, while preserving aromatase expression in granulosa cells (197). Moreover, antioxidant and mitochondrial effects of melatonin in ovarian tissue directly improve local steroidogenic efficiency, shifting the hormonal milieu toward estrogenic dominance compatible with ovulatory function (229). When combined, the genomic modulation of vitamin D, insulin sensitization of MI, and redox stabilization of melatonin collectively recalibrate the ovarian steroidogenic environment, attenuating androgen excess at multiple regulatory nodes.

#### 6.3.3 Ovulatory regulation and neuroendocrine synchrony

The ovulatory defect in PCOS stems from a composite disturbance of HPO signaling, local OS, and altered follicular energetics (7). Vitamin D participates in oocyte maturation through genomic control of calcium-dependent meiotic progression and improved endometrial receptivity via HOXA10 expression (230). MI contributes to restoration of LH/FSH pulsatility and oocyte competence by optimizing insulin and metabolic cues that drive granulosa-cell proliferation and estradiol synthesis (147). Clinical trials indicate higher ovulation rates and improved oocyte quality following MI supplementation, particularly in insulin-resistant PCOS subtypes (21). Melatonin, acting as a chronobiotic regulator of the HPO axis, synchronizes LH surge timing and enhances oocyte mitochondrial integrity through direct follicular uptake. Its nocturnal signaling resets circadian rhythms disrupted by metabolic stress, reinforcing the cyclicity essential for follicular dominance and luteinization (229). Thus, these bioactives converge at the redox-neuroendocrine interface: vitamin D modulates genomic readiness, MI enhances metabolic efficiency, and melatonin reinstates circadian alignment, each indispensable for the restoration of physiological ovulation.

### 6.4 Crosstalk with gut microbiota and adipokine networks

The gut-endocrine-ovarian axis has emerged as a critical interface linking metabolic, inflammatory, and reproductive disturbances in PCOS. Dysbiosis in PCOS is characterized by reduced α-diversity, an altered Firmicutes/Bacteroidetes ratio, and depletion of short-chain fatty acid (SCFA)-producing taxa (231). These shifts compromise intestinal barrier integrity, promote metabolic endotoxemia, and activate toll-like receptor-driven oxidative and inflammatory cascades that propagate IR and HA. Within this milieu, vitamin D, MI, and melatonin converge on microbial and adipokine pathways that jointly regulate redox and endocrine balance.

Vitamin D acts as a mucosal gatekeeper. Through VDR signaling in enterocytes and immune cells, it preserves tight junction architecture and suppresses TLR4/NF-κB activation, thereby limiting lipopolysaccharide-driven inflammation (232). Supplementation has been associated with enrichment of beneficial genera such as *Akkermansia muciniphila* and *Lactobacillus*, enhancing SCFA availability. SCFAs activate PPARγ and favor adiponectin secretion, improving insulin sensitivity and dampening inflammatory tone (233). In vitamin D-deficient PCOS phenotypes, repletion improves adiponectin/leptin ratios and lowers circulating TNF-α and IL-6, reflecting stabilization of the adipokine-microbiota-insulin axis (234). MI functions as both a gut-modulated metabolite and an insulin sensitizer. Bidirectional interactions with *Bifidobacterium* and *Lactobacillus* suggest that dysbiosis may impair MI bioavailability, exacerbating IR and androgen excess (235). MI supplementation restores PI3K/Akt signaling, lowers circulating insulin, and indirectly reshapes the microbial niche by reducing luminal glucose and OS (236). These effects favor SCFA-producing taxa and attenuate LPS-mediated adipose inflammation. Concurrently, MI increases adiponectin while reducing leptin and resistin, improving adipose redox balance and normalizing gonadotropin-androgen output (237). Moreover, melatonin serves as a circadian and microbial synchronizer (177). By aligning microbial gene expression involved in SCFA and bile acid metabolism, it coordinates nutrient handling with hormonal rhythms. In PCOS models, melatonin improves barrier integrity, suppresses epithelial OS, and reduces endotoxemia, promoting an eubiotic state enriched in butyrate producers such as *Faecalibacterium prausnitzii* and *Roseburia* (238). Via AMPK and SIRT1 signaling, melatonin enhances adiponectin and restrains leptin hypersecretion, improving metabolic flexibility (239).

Thus, vitamin D, MI, and melatonin intersect at NF-κB, AMPK, and PPARγ hubs, countering the triad of dysbiosis, inflammation, and endocrine disruption (240). Although early combination studies suggest improvements in IR, lipid profiles, and oxidative markers alongside favorable microbial shifts (218), robust trials with direct microbiome and adipokine endpoints are needed. Integrative multi-omics approaches may identify responder phenotypes, optimize circadian-aligned dosing, and advance systems-level nutraceutical strategies to restore homeostasis in PCOS.

## 7 Conclusions and future direction toward bioactive cocktails in PCOS

Evidence supports a mechanism-driven combination of vitamin D, MI, and melatonin to target the redox-endocrine circuitry that sustains PCOS. Each bioactive addresses a distinct yet intersecting node of dysfunction, i.e., vitamin D attenuates NF-κB-mediated inflammation and enhances insulin signaling through VDR-dependent transcriptional control, MI restores inositolphosphoglycan second-messenger activity, normalizing PI3K/Akt signaling and reducing hyperinsulinemia, and melatonin stabilizes mitochondrial function, activates Nrf2-dependent antioxidant defenses, and re-aligns circadian regulation of the HPO axis. When considered together, randomized trials and meta-analyses indicate improvements in ovulatory function, oocyte and embryo quality, insulin resistance indices, and oxidative-inflammatory biomarkers. These effects are often modest in isolation, reinforcing the rationale for coordinated, multi-level intervention. The biological logic for such ‘cocktails’ is compelling. Hyperinsulinemia amplifies androgen synthesis, OS disrupts granulosa mitochondrial competence and aromatase activity, and circadian misalignment perturbs gonadotropin timing. A layered regimen can address these pressures simultaneously: MI uncouples insulin-androgen crosstalk, vitamin D recalibrates inflammatory and steroidogenic set-points, and melatonin restores mitochondrial resilience and temporal order. Early combination studies suggest additive benefits with favorable safety profiles and high patient acceptability. Nonetheless, translation into standardized care requires rigor. Current gaps include limited factorial trials, short follow-up, under-reporting of live births, and insufficient phenotype stratification. Future studies should verify biochemical repletion, standardize dosing and timing, and integrate biomarker-anchored, systems-level endpoints.

To enhance the practical applicability of this framework, future research should also address the heterogeneity in dosing, treatment duration, and patient phenotypes observed across existing studies. Based on currently reported ranges, MI is commonly used at 2–4 g/day, melatonin at 2–5 mg/day (typically administered nocturnally), and vitamin D dosing is generally adjusted according to baseline deficiency status. Treatment duration in most studies ranges from 8 to 24 weeks, although shorter protocols are often applied in ART settings. Importantly, a phenotype-oriented approach may improve clinical relevance, where MI may be prioritized in insulin-resistant individuals, vitamin D in deficient or inflammatory phenotypes, and melatonin in patients with pronounced oxidative stress or circadian disruption. While these considerations remain provisional and require validation, they provide a structured direction for future trial design and help move this concept toward a more individualized, redox-guided strategy. If validated, this triad could redefine PCOS management as a precision nutraceutical strategy, shifting therapy from fragmented symptom control to restoration of redox-endocrine homeostasis.

Moreover, this framework should evolve toward a more individualized and practical approach that considers both patient variability and external influences. PCOS is not a uniform condition, and in some patients, adrenal androgen excess or altered redox status may play a more dominant role than insulin resistance. This highlights the need for future studies to move beyond broad categories and instead use hormonal, metabolic, and oxidative markers to better guide treatment choices. In addition, the role of gut health deserves more attention, as imbalances in microbiota can reduce nutrient absorption and sustain inflammation, potentially limiting the effectiveness of these bioactives. Supportive strategies such as diet-based interventions and microbiota-focused approaches may therefore enhance outcomes. Importantly, this combination should not be seen as a replacement for lifestyle modification. Regular physical activity, stress management, and mind-body practices such as yoga may help lower oxidative stress and improve endocrine balance, thereby supporting the actions of melatonin and related pathways. Attention to micronutrient status is also important, as elements like zinc, selenium, and chromium are required for optimal antioxidant enzyme function. Altogether, combining these factors with the proposed bioactive approach may help move toward a more personalized and clinically meaningful strategy in PCOS.

## Glossary

ΔΨm: Mitochondrial membrane potential
25(OH)D: 25-Hydroxyvitamin D
3β-HSD: 3-beta-Hydroxysteroid dehydrogenase
4-HNE: 4-hydroxynonenal
8-OHdG: 8-hydroxy-2’-deoxyguanosine
ADMA: Asymmetric dimethylarginine
AGE: Advanced glycation end products
AMH: Anti-Müllerian hormone
AMPK: Adenosine monophosphate-activated protein kinase
ART: Assisted reproductive technology
CAT: Catalase
CoA: Coenzyme A
Cyp: Cytochrome
DCI: D-chiro-inositol
DHEA: Dehydroepiandrosterone
DHT: Dihydrotestosterone
ELISA: Enzyme-linked immunosorbent assay
ETC: Electron transport chain
FSH: Follicle-stimulating hormone
GF: Graafian follicle
GH: Growth Hormone
GLUT-4: Glucose transporter type 4
GPx: Glutathione peroxidase
GR: Glutathione reductase
GRAS: Generally recognized as safe
GSH: Glutathione
GSH/GSSG: Reduced-to-oxidized glutathione
HA: Hyperandrogenism
HbA1c: Glycated hemoglobin
HDL: High-density lipoprotein
HPG: Hypothalamic–pituitary-gonadal
HPO: Hypothalamic-pituitary-ovarian
hs-CRP: High-sensitivity C-reactive protein
ICSI: Intracytoplasmic sperm injection
IL-10: Interleukin-10
IL-17: Interleukin-17
IL-6: Interleukin-6
IPG: Inositolphosphoglycan
IR: Insulin resistance
IRS: Insulin receptor substrate
IVF: *In vitro* fertilization
LDL: Low-density lipoprotein
LH: Luteinizing hormone
LPO: Lipid peroxidation
MAPK: Mitogen-activated protein kinase
MDA: Malondialdehyde
MI: Myo-inositol
MII: Metaphase II
MP: Multilaminar primary follicle
mtDNA: Mitochondrial DNA
NADPH: Nicotinamide adenine dinucleotide phosphate
NF-κB: Nuclear factor-kappa B
NMRI: Naval Medical Research Institute
NO: Nitric oxide
NOS: Nitric oxide synthase
NOX: NADPH oxidase
NRF: Nuclear related factor
OCP: Oral Contraceptive Pill
OS: Oxidative stress
P: Primordial follicle
PCOS: Polycystic ovary syndrome
PI3K: Phosphatidylinositol 3-kinase
PPARγ: Peroxisome proliferator-activated receptor gamma
p-STAT3: Phosphorylated signal transducer and activator of transcription 3
PUFA: Polyunsaturated fatty acid
RAS: Renin angiotensin system
ROS: Reactive oxygen species
RXR: Retinoid X receptor
SCFA: Short-chain fatty acid
SCN: Suprachiasmatic nucleus
SF: Secondary follicle
SHBG: Sex hormone-binding globulin
SOD: Superoxide dismutase
StAR: Steroidogenic acute regulatory
TAC: Total antioxidant capacity
TNFα: Tumor necrosis factor alpha
TSH: Thyroid-stimulating hormone
UP: Uilaminar primary follicle
FDA: United States Food and Drug Administration
VDR: Vitamin D receptor
VDRE: Vitamin D response elements
VLDL: Very low-density lipoprotein
WHO: World Health Organization
